# Maternal Fc-mediated non-neutralizing antibody responses correlate with protection against congenital human cytomegalovirus infection

**DOI:** 10.1172/JCI156827

**Published:** 2022-08-15

**Authors:** Eleanor C. Semmes, Itzayana G. Miller, Courtney E. Wimberly, Caroline T. Phan, Jennifer A. Jenks, Melissa J. Harnois, Stella J. Berendam, Helen Webster, Jillian H. Hurst, Joanne Kurtzberg, Genevieve G. Fouda, Kyle M. Walsh, Sallie R. Permar

**Affiliations:** 1 Medical Scientist Training Program, Department of Molecular Genetics and Microbiology and; 2 Duke Human Vaccine Institute, Duke University, Durham, North Carolina, USA.; 3 Duke Children’s Health & Discovery Initiative, Duke University, Durham, North Carolina, USA.; 4 Department of Pediatrics, Weill Cornell School of Medicine, New York, New York, USA.; 5 Department of Neurosurgery and; 6 Department of Pediatrics, Duke University, Durham, North Carolina, USA.; 7 Carolinas Cord Blood Bank, Duke University Medical Center, Durham, North Carolina, USA.

**Keywords:** Immunology, Infectious disease, Adaptive immunity, Immunoglobulins

## Abstract

Human cytomegalovirus (HCMV) is the most common congenital infection and a leading cause of stillbirth, neurodevelopmental impairment, and pediatric hearing loss worldwide. Development of a maternal vaccine or therapeutic to prevent congenital HCMV has been hindered by limited knowledge of the immune responses that protect against HCMV transmission in utero. To identify protective antibody responses, we measured HCMV-specific IgG binding and antiviral functions in paired maternal and cord blood sera from HCMV-seropositive transmitting (*n =* 41) and non-transmitting (*n =* 40) mother-infant dyads identified via a large, US-based, public cord blood bank. We found that high-avidity IgG binding to HCMV and antibody-dependent cellular phagocytosis (ADCP) were associated with reduced risk of congenital HCMV infection. We also determined that HCMV-specific IgG activation of FcγRI and FcγRII was enhanced in non-transmitting dyads and that increased ADCP responses were mediated through both FcγRI and FcγRIIA expressed on human monocytes. These findings suggest that engagement of FcγRI/FcγRIIA and Fc effector functions including ADCP may protect against congenital HCMV infection. Taken together, these data can guide future prospective studies on immune correlates against congenital HCMV transmission and inform HCMV vaccine and immunotherapeutic development.

## Graphical abstract

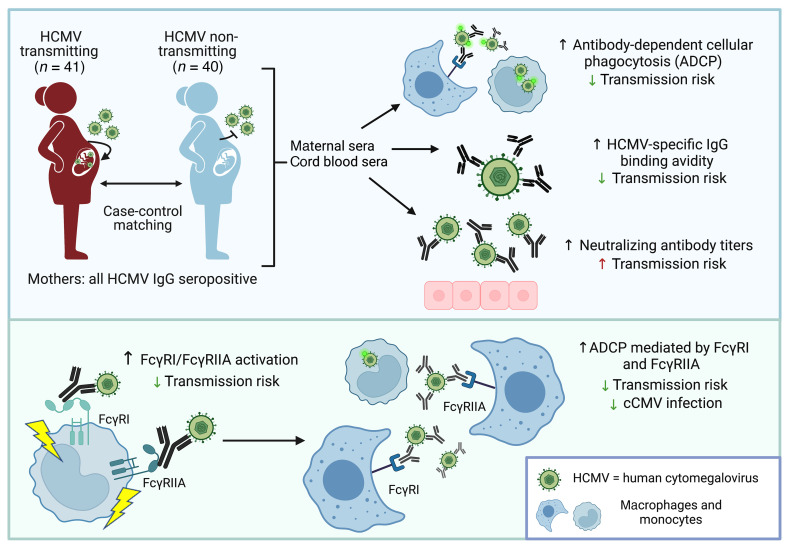

## Introduction

Human cytomegalovirus (HCMV) is the most common congenital infection worldwide, affecting 1 of 200 births or nearly 1 million newborns annually (1, 2). Most congenital HCMV (cCMV) infections are asymptomatic, yet serious disease outcomes can occur, including stillbirth, intrauterine growth restriction, neonatal multi-organ disease, neurodevelopmental impairment, and sensorineural hearing loss (3, 4) Moreover, cCMV infection has recently been linked to an elevated risk of acute lymphoblastic leukemia (5–7). Newborn screening for and public awareness of cCMV remain limited, leaving most cases undiagnosed and the true burden of disease underestimated (5, 8, 9). There are no licensed vaccines or therapeutics to prevent cCMV, and an improved understanding of protective immunity against cCMV transmission is urgently needed to guide novel interventions.

HCMV is a ubiquitous, host-restricted β-herpesvirus with multiple envelope glycoproteins and complexes, including glycoprotein B (gB) and gH/gL “dimer”; these can associate with gO to form the gH/gL/gO “trimer” or pUL128/130/131 to form the “pentamer” complex (10). HCMV envelope glycoproteins mediate viral entry, and following primary infection, the host remains latently infected for life (10–12). Over 80% of women of reproductive age worldwide are latently infected with HCMV, and congenital transmission occurs in maternal primary and nonprimary infection (i.e., reactivation from latency or reinfection with new strains) (2, 12–16). Mothers with primary infection have a 30% risk of fetal transmission, whereas those with nonprimary infection have a 1%–4% risk (2, 17–19), suggesting that preexisting maternal immunity partially protects against cCMV. In maternal primary infection, high-avidity IgG binding to HCMV and antipentamer IgG levels are correlated with decreased congenital transmission risk (20–23). However, in maternal nonprimary infection, protective immunity remains unclear, as HCMV-specific IgG levels and neutralizing antibody titers do not always correlate with reduced congenital transmission (24–26).

Identifying protective immune responses in maternal primary and nonprimary infection is necessary for developing effective interventions to prevent cCMV. High-avidity HCMV-specific IgG binding and neutralizing antibodies have been the main targets of vaccines and therapeutics (10, 27). Yet maternal treatment with HCMV hyperimmune globulin — a pooled preparation of high-avidity, neutralizing antibodies — to prevent fetal transmission following primary infection has had limited efficacy (28–33).

Emerging evidence indicates that non-neutralizing antibody functions also protect against HCMV infection, but to our knowledge, these have not been targeted in HCMV vaccines or immunotherapeutics (34–37). Moreover, whether Fc-mediated non-neutralizing antibody functions (e.g., antibody-dependent cellular phagocytosis [ADCP] and antibody-dependent cellular cytotoxicity [ADCC]) protect against cCMV has not been explored. In this study, we focused on ADCP, since vaccine trials suggest that Fc effector functions independent of ADCC mediate protection against HCMV (34, 38). We hypothesized that ADCP may protect against cCMV infection, as ADCP can eliminate virus:IgG immune complexes and virally infected cells; and this could prevent systemic maternal viral replication, dissemination, and transmission across the maternal-fetal interface (39).

To identify protective immune responses against cCMV transmission, we compared antibody profiles in HCMV-seropositive transmitting and non-transmitting mother-infant dyads identified as donors to a US-based public cord blood bank. In our primary analysis, we compared 13 predefined IgG binding, neutralizing, and non-neutralizing antibody responses in transmitting versus non-transmitting women. In an exploratory analysis, we used systems serology to define differences between transmitting and non-transmitting dyads and examined the role of Fc-mediated immunity in cCMV transmission. Insights from this study could inform vaccine and therapeutic development to prevent cCMV infection, a major cause of perinatal and pediatric morbidity worldwide.

## Results

### Baseline characteristics of HCMV transmitting and non-transmitting mother-infant dyads.

Our study included sera from 81 mother-infant dyads identified retrospectively as donors to the Carolinas Cord Blood Bank (CCBB), a large US-based public cord blood bank (Supplemental Figure 1; supplemental material available online with this article; https://doi.org/10.1172/JCI156827DS1). cCMV infection was defined based on the presence of HCMV viremia in the donated cord blood plasma. Forty-one dyads with cCMV infection (“HCMV transmitting”) were matched to 40 dyads with HCMV IgG–seropositive mothers who gave birth to cCMV-uninfected infants (“HCMV non-transmitting”). Matching criteria included infant sex, infant race, maternal age, and delivery year. Only women with healthy, uncomplicated pregnancies that gave birth at term were included in our study; cord blood donors were screened for signs of (a) neonatal sepsis, (b) congenital infection (petechial rash, thrombocytopenia, hepatosplenomegaly), and (c) congenital abnormalities. Demographic and clinical characteristics were comparable between transmitting and non-transmitting dyads, though cCMV cases had a nonsignificantly higher rate of Cesarean section (56% vs. 40%, Fisher’s exact *P* = 0.22; Table 1).

To assess whether mothers may have had primary or nonprimary HCMV infection during pregnancy, we measured HCMV-specific IgG avidity and IgM in maternal sera collected at delivery (40). HCMV IgG avidity indexes were similar in transmitting and non-transmitting mothers (Table 1). Yet 11 of 41 (26.8%) of transmitting mothers had detectable HCMV-specific IgM compared with only 2 of 40 (5%) of non-transmitting mothers (Table 1). These data suggest that during pregnancy transmitting women likely had a higher rate of primary infection or reinfection, which are known risk factors for congenital transmission (40, 41). To further assess comparability between groups, we quantified HCMV viral loads in maternal sera and found that a similar proportion of transmitting (26.8%) and non-transmitting (37.5%) women had low-level HCMV DNAemia (Table 1), a finding similar to that in healthy HCMV-seropositive women (42).

### Maternal and cord blood sera from HCMV transmitting dyads have high HCMV-specific IgG levels.

**Figure 1:**
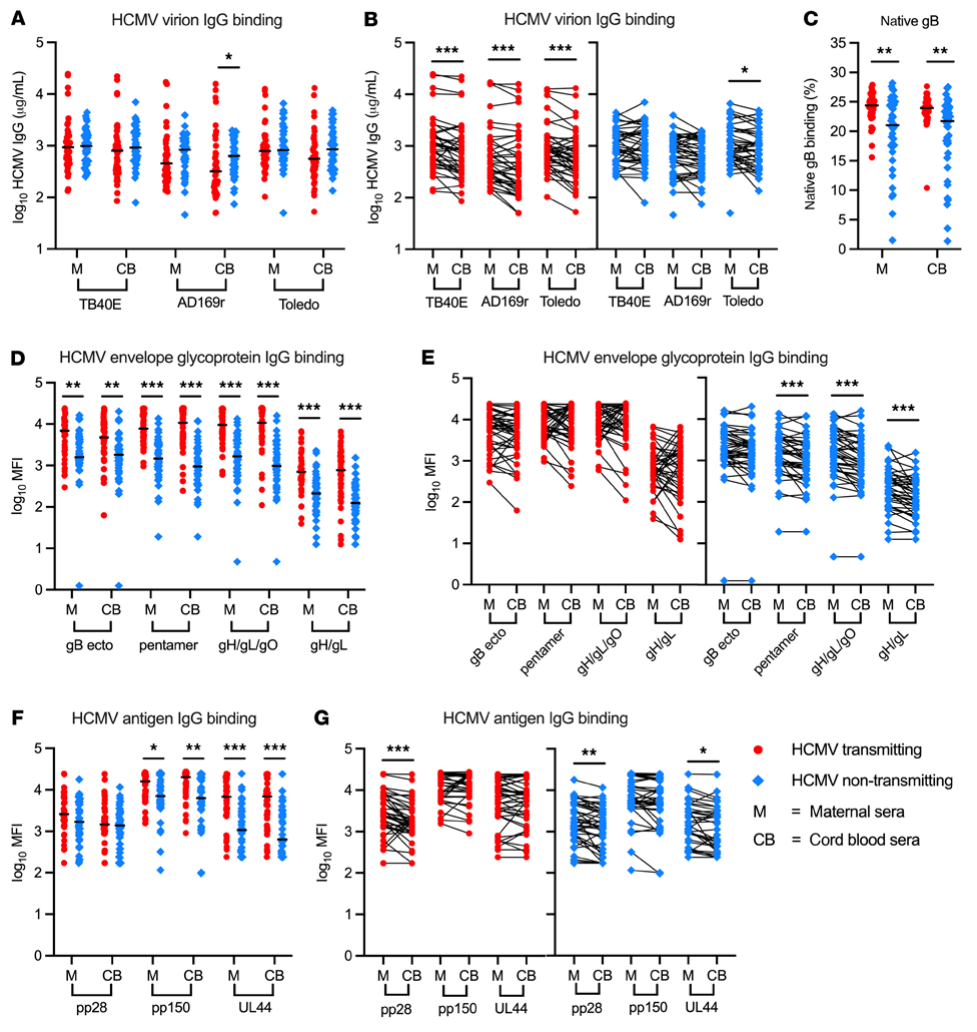
HCMV-specific IgG binding and transplacental IgG transfer in HCMV transmitting and non-transmitting mother-infant dyads. HCMV-specific IgG levels against the HCMV strains TB40E, AD169r, and Toledo were measured using ELISA. IgG binding to cell-associated gB was quantified using a flow-based assay with HEK293T cells transfected with full-length gB. HCMV antigen IgG binding was measured using a Luminex-based BAMA and reported as MFI. IgG binding responses in maternal (M) and cord blood (CB) sera were compared between and within HCMV transmitting (red circles, *n* = 41) and non-transmitting (blue diamonds, *n* = 40) mother-infant dyads. (**A** and **B**) IgG binding to HCMV virus antigens (**A**) in transmitting versus non-transmitting dyads and (**B**) within paired maternal and cord blood sera. (**C**) IgG binding to cell-associated gB in transmitting versus non-transmitting dyads. (**D** and **E**) IgG binding to HCMV envelope glycoproteins (**D**) in transmitting versus non-transmitting dyads and (**E**) within paired maternal and cord blood sera. (**F** and **G**) IgG binding to HCMV antigens (**F**) in transmitting versus non-transmitting dyads and (**G**) within paired maternal and cord blood sera. gB ecto, gB ectodomain. Horizontal black bars denote median. FDR-corrected *P* values reported for Mann-Whitney *U* test (**A**, **C**, **D**, and **F**) and Wilcoxon’s signed-rank test (**B**, **E**, and **G**). *P < 0.05, **P < 0.01, ***P < 0.001.

We first quantified IgG binding against 3 distinct HCMV strains: TB40E (an endotheliotropic strain expressing pentamer), AD169r (a laboratory-adapted strain with repaired pentamer expression), and Toledo (a low-passage clinical isolate lacking pentamer) (Supplemental Figure 2, A–D). Median IgG binding against each HCMV strain was similar in transmitting and non-transmitting groups, except in the case of cord blood IgG binding to AD169r, which was lower in infected infants (Figure 1A). Within transmitting dyads, whole virus IgG binding was lower in cord blood than maternal sera across all strains (Figure 1B). However, IgG binding against envelope glycoproteins, including gB, pentamer, gH/gL/gO, and gH/gL, was significantly elevated (2.5- to 10-fold) in transmitting versus non-transmitting dyads, and glycoprotein-specific IgG was efficiently transferred into cord blood of infected infants (Figure 1, C–E). These data reveal that infants with cCMV infection received high levels of maternal HCMV-specific IgG via placental antibody transfer. To explore whether other HCMV-specific antibodies were associated with protection, we measured IgG binding against the HCMV tegument proteins pp28 and pp150 and the viral replication factor UL44, which are known to elicit potent IgG responses. IgG binding to pp150 and UL44 was also higher in transmitting versus non-transmitting dyads, with some differences within dyads (Figure 1, F and G). Together, these findings indicate that a higher quantity of HCMV-specific IgG at the delivery time point is not correlated with lower cCMV transmission risk.

### HCMV non-transmitting women have higher relative IgG binding to whole virus antigen and cell-associated gB.

In our recent study on placental IgG transfer in cCMV infection (43), we observed that HCMV transmitting women had elevated total IgG levels (i.e., hypergammaglobulinemia). In this larger cohort, we also found that total IgG levels were higher in transmitting versus non-transmitting women (Supplemental Figure 3A). When we adjusted for total IgG levels, IgG binding against pentamer, gH/gL/gO, gH/gL, and UL44 remained higher in transmitters, but non-transmitters had higher relative IgG binding to whole-virus antigens and cell-associated gB (Supplemental Figure 3, B and C). These data indicate that IgG binding to gB expressed in the native conformation on a virion or infected cell surface and other HCMV antigens not captured in our study may be associated with reduced cCMV transmission risk.

### HCMV-specific IgG binding avidity is increased in non-transmitting versus transmitting dyads.

**Figure 2:**
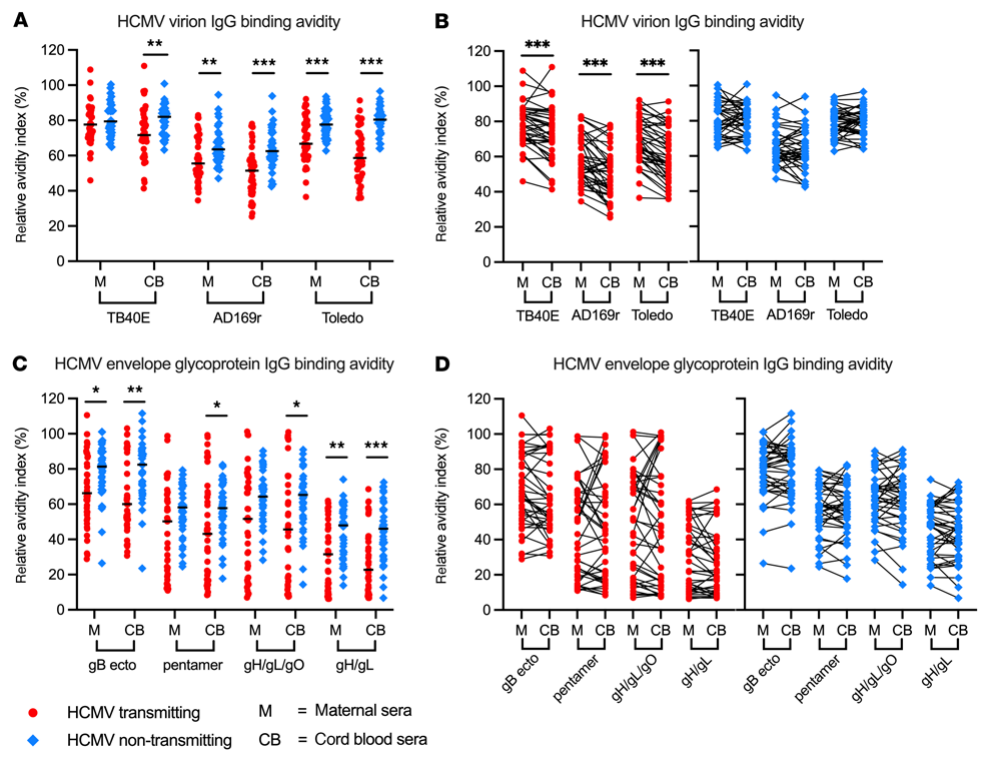
HCMV-specific IgG binding avidity is increased in non-transmitting versus transmitting mother-infant dyads. HCMV-specific IgG binding avidity against HCMV strains TB40E, AD169r, and Toledo were measured using whole-virion ELISA with an additional dissociation step using urea, and RAI was calculated as (OD with urea/OD without urea) × 100%. HCMV glycoprotein–specific IgG binding avidity was measured using a Luminex-based BAMA with an additional dissociation step with sodium citrate and RAI was calculated as (MFI with sodium citrate/MFI with 1× PBS) × 100%. IgG binding avidity in maternal and cord blood sera was compared between and within HCMV transmitting (red circles, *n* = 41) and non-transmitting (blue diamonds, *n* = 40) mother-infant dyads. (**A** and **B**) Whole-virus HCMV-specific IgG binding avidities (**A**) in transmitting versus non-transmitting dyads and (**B**) within paired maternal and cord blood sera. (**C** and **D**) HCMV glycoprotein–specific IgG binding avidity (**C**) in transmitting versus non-transmitting dyads and (**D**) within paired maternal and cord blood sera. Horizontal black bars denote median. FDR-corrected *P* values reported for Mann-Whitney *U* test (**A** and **C**) and Wilcoxon’s signed-rank test (**B** and **D**). *P < 0.05, **P < 0.01, ***P < 0.001.

We next assessed the quality of HCMV-specific IgG in transmitters versus non-transmitters by measuring IgG binding avidity. Maternal sera from non-transmitters had higher-avidity IgG binding to AD169r and Toledo, but not TB40E, and cord blood IgG binding avidity was increased across all strains in uninfected versus infected infants (Figure 2A). Within dyads, whole-virus IgG binding avidity was lower in paired cord blood versus maternal sera in transmitting but not non-transmitting dyads (Figure 2B). HCMV glycoprotein–specific IgG binding avidity was also lower in the cord blood of infected versus uninfected infants, though no significant differences were observed within dyads (Figure 2, C and D). In a sensitivity analysis excluding mothers with detectable HCMV-specific IgM as a surrogate biomarker for recent primary infection or reinfection, many of these avidity differences persisted. Non-transmitting dyads still had higher avidity IgG binding to HCMV, and low-avidity HCMV-specific IgG was enriched in the cord blood of infected infants (Supplemental Figure 4, A–C). Together, these findings suggest that high-avidity HCMV-specific IgG in the maternal and fetal circulation is associated with protection against cCMV infection, even when excluding mothers with recent primary infection or reinfection.

### Neutralizing and non-neutralizing antibody functions in transmitting and non-transmitting dyads.

**Figure 3:**
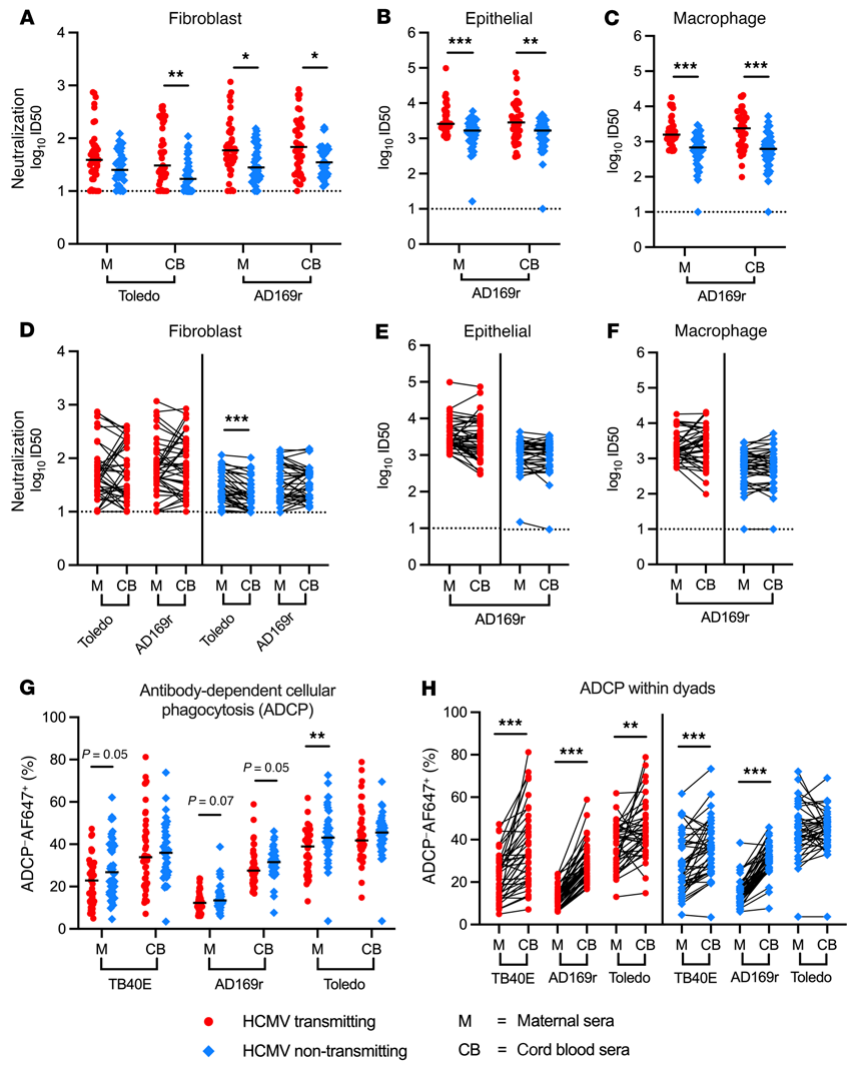
Neutralizing and non-neutralizing antibody responses differ in HCMV transmitting compared with non-transmitting mother-infant dyads. Functional antiviral antibody responses in maternal and cord blood sera were compared between and within HCMV transmitting (red circles, *n* = 41) and non-transmitting (blue diamonds, *n* = 40) mother-infant dyads. Neutralization was measured by HCMV IE1 staining, and titers were calculated as ID_50_, equivalent to the serum dilution that inhibited 50% of the maximum infection in virus-only wells. (**A**–**C**) Neutralization titers against HCMV strains Toledo and/or AD169r in (**A**) fibroblasts (HFFs), (**B**) epithelial cells (ARPEs), and (**C**) macrophages (differentiated THP-1 cells) in transmitting versus non-transmitting dyads; and (**D**–**F**) within paired maternal and cord blood sera. ADCP of AF647 fluorophore–conjugated HCMV virions by THP-1 monocytes was quantified using a flow-based assay and calculated as percentage AF647-positive cells. (**G** and **H**) HCMV-specific ADCP (**G**) in transmitting versus non-transmitting dyads and (**H**) within paired maternal and cord blood sera. Horizontal black bars denote median. Dotted lines indicate the lower limit of detection (ID_50_ = 10). FDR-corrected *P* values reported for Mann-Whitney *U* test (**A**–**C** and **G**) and Wilcoxon’s signed-rank test (**D**–**F** and **H**). *P < 0.05, **P < 0.01, ***P < 0.001.

Next, we compared neutralizing and non-neutralizing antibody functions in transmitting and non-transmitting dyads (Supplemental Figure 5, A–D). Neutralizing antibody titers were 1.5- to 4-fold higher in transmitting versus non-transmitting dyads across strains and cell types (Figure 3, A–C). Within dyads, neutralizing titers were mostly similar in paired cord blood and maternal sera (Figure 3, D–F). These data indicate that HCMV-neutralizing antibodies are effectively transferred across the placenta regardless of transmission status. In contrast, HCMV-specific ADCP, a non-neutralizing antibody response, was higher in non-transmitting versus transmitting women (Figure 3G). This difference was significant for Toledo (*P* = 0.0057, FDR-corrected *P* = 0.011), with a trend toward increased ADCP for TB40E (*P =* 0.053) and AD169r (*P =* 0.068), which may not have reached statistical significance due to the lower overall ADCP measured against these strains (Supplemental Figure 5, E and F). Within dyads, ADCP was highly enriched in paired cord blood versus maternal sera (Figure 3H). ADCP may have been higher in fetal than maternal circulation because fewer inhibitory factors, such as IgA, were present; or because of enhanced placental transfer of ADCP-mediating IgG, a phenomenon that has been observed for ADCC-eliciting antibodies (44). Overall, these data suggest that non-neutralizing antibody responses may be important for preventing cCMV.

### ADCP and high-avidity IgG binding to HCMV correlate with decreased risk of cCMV infection.

For our primary analysis, we hypothesized that 13 maternal antibody responses would be correlated with reduced risk of cCMV infection (Table 2). Using univariate logistic regression, we found that 12 of the 13 variables were significantly associated with cCMV transmission risk. However, high-magnitude IgG binding to HCMV envelope glycoproteins and neutralization were associated with increased risk, whereas high-avidity IgG binding and ADCP were associated with decreased risk (Table 2). After adjusting for maternal total IgG and HCMV-specific IgM, we found that HCMV glycoprotein-specific IgG binding and neutralization were still associated with increased risk, but IgG binding avidity was no longer significantly associated with reduced risk (Supplemental Table 1). ADCP against Toledo remained significantly associated with protection against cCMV transmission in both the adjusted univariate regression models (Supplemental Table 1).

As many immune variables in our predefined primary analysis were strongly correlated, we used the least absolute shrinkage and selection operator method (LASSO) for feature selection prior to multivariable analysis. LASSO is an approach to minimize overfitting a regression model that shrinks the coefficients of poorly predictive variables to zero, thereby removing them from the model. First, the cohort was randomly split into training and test data sets, and a 5-fold nested cross-validation with 5 repeats was used to train the LASSO model. LASSO-selected features included magnitude of pentamer IgG binding, avidity of gB IgG binding, avidity of gH/gL/gO IgG binding, and ADCP against the Toledo strain (Table 2). Higher pentamer IgG binding was associated with increased risk, whereas higher ADCP and IgG binding avidity were associated with decreased risk of cCMV infection in this multivariable model using the LASSO-selected features. In the out-of-sample test data, this 4-parameter LASSO model had an accuracy of 0.75 (95% CI, 0.48–0.93) in predicting cCMV transmission risk, with 1.00 equaling perfect prediction and 0.45 equaling the random prediction rate after class label permutation (Supplemental Figure 6).

**Figure 4:**
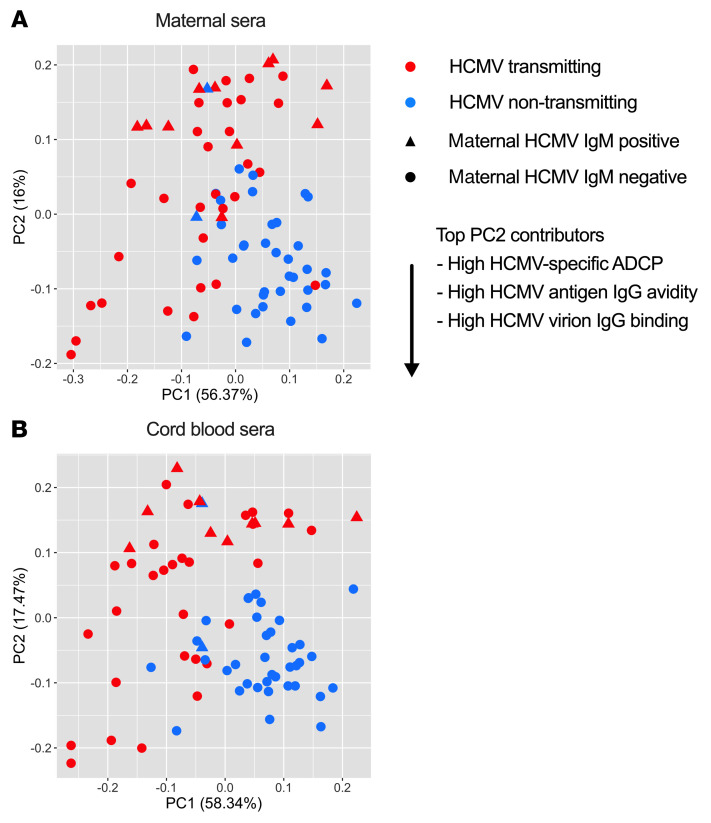
PCA highlights distinct HCMV-specific antibody responses in HCMV transmitting versus non-transmitting dyads. PCA across antibody responses in HCMV transmitting (red, *n* = 41) and non-transmitting (blue, *n* = 40) mother-infant dyads. Triangles (*n* = 14) indicate dyads in which mothers screened positive for HCMV-specific IgM responses, and circles (*n* = 67) indicate dyads in which mothers had no detectable HCMV-specific IgM responses. Scatterplot of PC1 and PC2 for (**A**) maternal and (**B**) cord blood sera.

Next, we used a systems serology approach leveraging principal components analysis (PCA) to explore differences in HCMV-specific antibody responses in transmitting versus non-transmitting dyads. PC1 accounted for 57% and 59% of the variance, respectively; however, PC2, which accounted for 16% and 17% of the variance, was superior at delineating between transmitting and non-transmitting groups (Figure 4, A and B). The top contributors to PC2 included ADCP against Toledo, TB40E, and AD169r; IgG binding avidity to HCMV glycoproteins; and IgG binding magnitude to the Toledo, TB40E, and AD169r strains (Figure 4). These PCA results further established that IgG binding to HCMV antigens distinct from the major envelope glycoproteins, high-avidity HCMV-specific IgG binding, and ADCP responses were enriched in the maternal and cord blood sera of non-transmitting compared with transmitting dyads.

### HCMV-specific IgG binding to FcγRI and FcγRII differs in transmitting and non-transmitting dyads.

**Figure 5:**
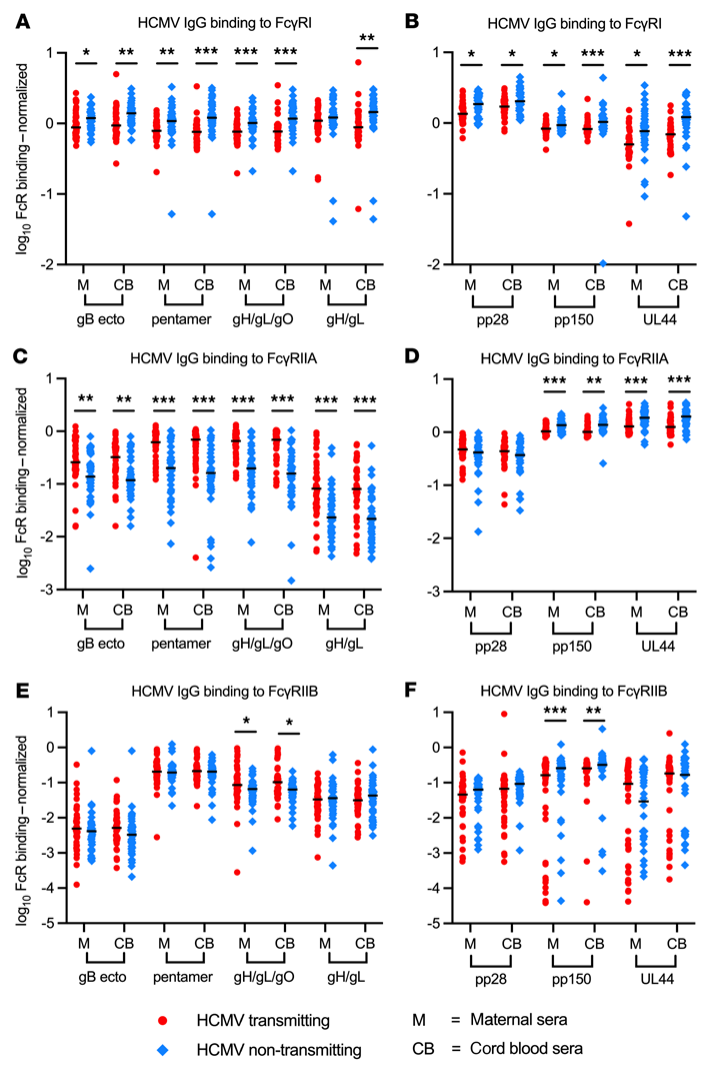
HCMV-specific IgG binding to FcγRI, FcγRIIA, and FcγRIIB differs in transmitting and non-transmitting dyads. HCMV antigen–specific IgG binding to FcγRs was measured using a Luminex-based BAMA with a biotinylated FcγR and streptavidin-PE detection antibody. HCMV antigen–specific IgG binding to host FcγRs was normalized as a ratio of total antigen-specific IgG binding and was compared between transmitting (red circles, *n* = 41) and non-transmitting (blue diamonds, *n* = 40) mother-infant dyads. (**A** and **B**) HCMV-specific IgG binding to activating FcγRI, (**C** and **D**) activating FcγRIIA (high-affinity H131 variant), and (**E** and **F**) inhibitory FcγRIIB. Horizontal black bars denote median. FDR-corrected *P* values reported for Mann-Whitney U test (**A**–**F**). *P < 0.05, **P < 0.01, ***P < 0.001.

After identifying ADCP as a potential correlate of protection, we sought to understand why ADCP was enhanced in non-transmitting dyads. We hypothesized that HCMV-specific IgG from non-transmitting dyads may better engage the host Fcγ receptors (FcγRs) on innate immune cells that mediate ADCP. To explore this hypothesis, we measured HCMV-specific IgG binding to FcγRI and FcγRII, including the activating FcγRIIA and inhibitory FcγRIIB (45–47) (Supplemental Figure 7). To compare FcγR binding between the groups, we normalized FcγR-specific IgG binding to total IgG binding to antigen-coated beads at baseline. Normalized HCMV-specific IgG binding to FcγRI was significantly higher in non-transmitting versus transmitting dyads (Figure 5, A and B) and highly enriched in the cord blood of uninfected infants (Supplemental Figure 8A). HCMV-specific IgG binding to FcγRI was also negatively correlated (*P* < 0.05) with HCMV viral loads in the cord blood of infected infants, suggesting that FcγRI engagement may help control viremia. Normalized gB-, pentamer-, gH/gL/gO-, and gH/gL-specific IgG binding to FcγRIIA was higher in transmitters, whereas pp28-, pp150-, and UL44-specific IgG binding to FcγRIIA was higher in non-transmitters (Figure 5, C and D, and Supplemental Figure 8B). Only gH/gL/gO- and pp150-specific IgG binding to FcγRIIB differed between groups, with the former higher in transmitters and the latter higher in non-transmitters (Figure 5, E and F). These findings suggest that engagement of FcγRI, and to a lesser extent FcγRIIA, may mediate protection against cCMV transmission, which we sought to explore further using a functional signaling assay.

### FcγRI and FcγRII activation is enhanced in non-transmitting dyads and correlated with ADCP.

**Figure 6:**
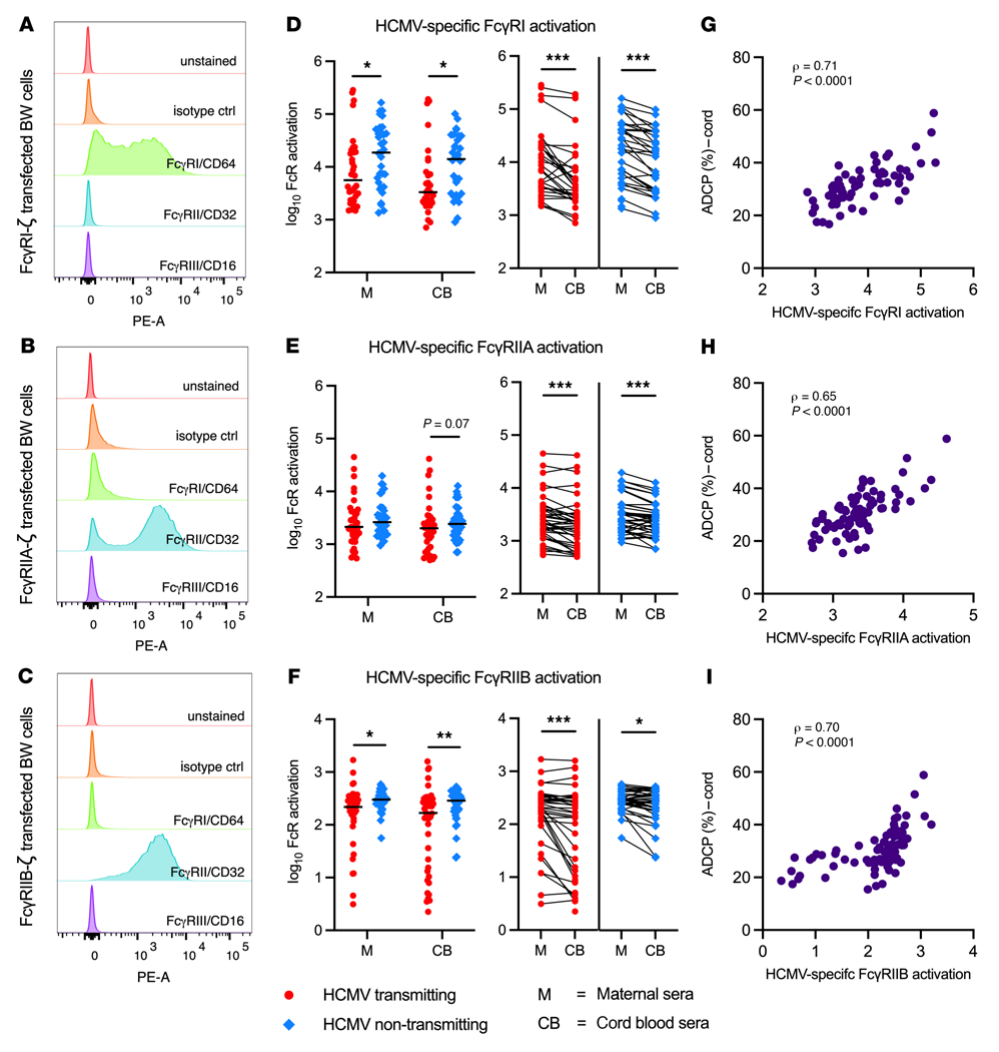
HCMV-specific IgG activation of FcγRI, FcγRIIA ,and FcγRIIB is increased in non-transmitting compared with transmitting mother-infant dyads. HCMV-specific IgG activation of FcγRs was measured using maternal and cord blood sera from HCMV transmitting (red circles, *n* = 41) and non-transmitting (blue diamonds, *n* = 40) mother-infant dyads. For quantification of HCMV-specific IgG activation of FcγRs, mouse BW cell lines stably expressing chimeric human FcγRs fused to a mouse CD3ζ signaling domain were co-cultivated with virus:serum immune complexes for 20 hours. Activation of FcγRs by immune complexes triggered CD3ζ signaling and mouse IL-2 secretion, which was measured by ELISA as a quantitative readout of HCMV-specific IgG signaling through host FcγRs. (**A**–**C**) Flow cytometry of BW cell lines including unstained cells, isotype control, anti-FcγRI/CD64, anti-FcγRII/CD32, and anti-FcγRIII/CD16 PE-conjugated antibodies. (**D**–**F**) HCMV-specific IgG activation of (**D**) FcγRIA, (**E**) FcγRIIA, (**F**) FcγRIIB in transmitting versus non-transmitting dyads and within paired maternal and cord blood sera. (**G**–**I**) Spearman’s correlations between HCMV-specific IgG FcγR activation and ADCP. Horizontal black bars denote median. *P* values for Mann-Whitney *U* test (**D**–**F** between groups) and Wilcoxon’s signed-rank test (**D**–**F** within dyads). *P < 0.05, **P < 0.01, ***P < 0.001.

To quantify HCMV-specific IgG activation of FcγRI and FcγRII, we used mouse BW thymoma cell lines expressing chimeric human FcγRs that secrete mouse IL-2 upon IgG engagement as a quantitative readout for FcγR activation (48, 49). We first confirmed that each BW cell line was expressing the FcγR of interest (Figure 6, A–C). HCMV-specific FcγRI activation was 3- to 4-fold higher in non-transmitting dyads (Figure 6D). There was also a trend toward higher FcγRIIA activation and significantly higher FcγRIIB activation in non-transmitting dyads (Figure 6, E and F). It has been hypothesized that increased IgG binding to activating versus inhibitory FcγRs improves antiviral phagocytosis, since preferential IgG binding to FcγRIIA over FcγRIIB has been correlated with enhanced ADCP in HIV infection (50, 51). Yet we found that the ratio of FcγRIIA to FcγRIIB IgG engagement did not correlate with ADCP or decreased cCMV transmission risk in our cohort (Supplemental Figure 9). Instead, higher FcγRI, FcγRIIA, and FcγRIIB activation all correlated with higher ADCP (*P* < 0.0001) (Figure 6, G–I, and Supplemental Figure 10). These data imply that engagement of host FcγRI and FcγRII may both help mediate effective ADCP of HCMV.

### ADCP of HCMV is mediated by FcγRI and FcγRIIA on human monocytes.

**Figure 7:**
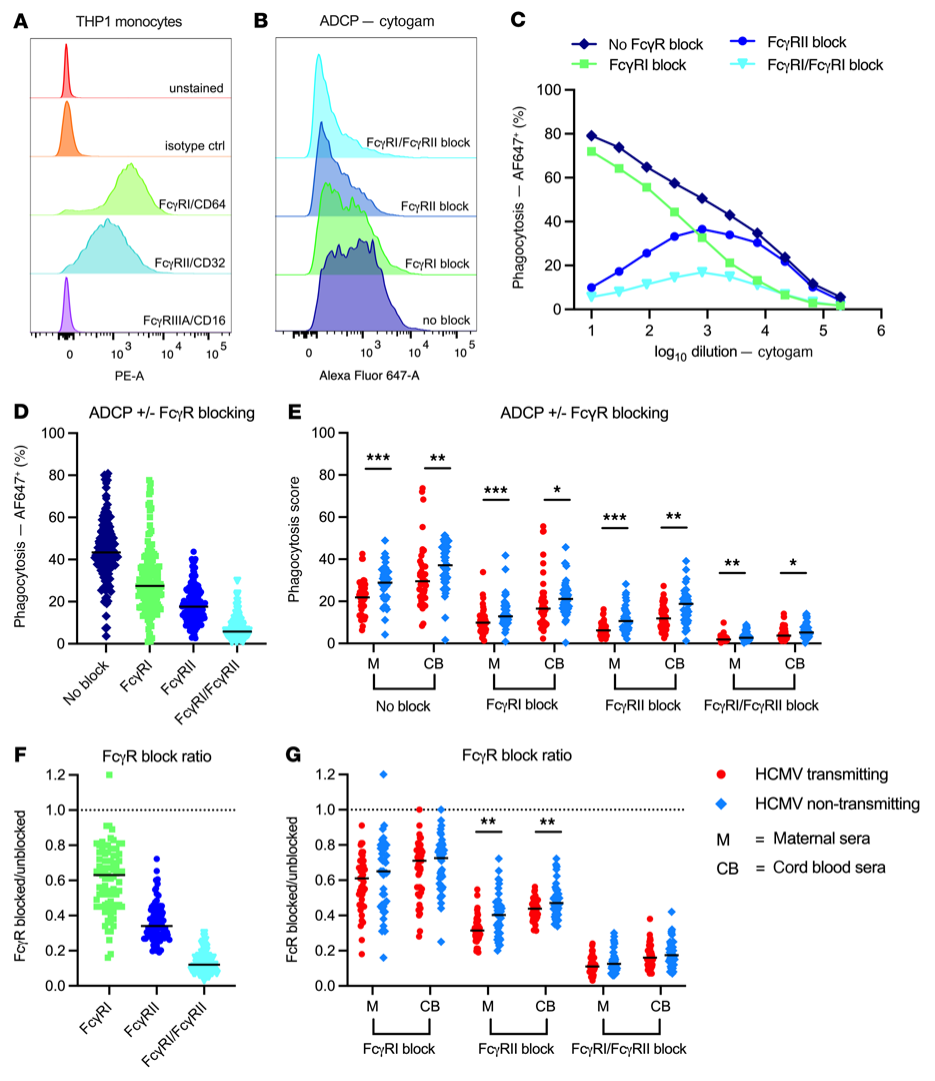
ADCP of HCMV is mediated by FcγRI and FcγRIIA expressed on human monocytes. ADCP of AF647 fluorophore–conjugated HCMV virions (Toledo strain) by THP-1 monocytes was quantified using a flow-based assay. In blocking experiments, THP-1 monocytes were preincubated with FcγR-blocking antibodies for 90 minutes prior being coincubation in virus:serum immune complexes. (**A**) Flow cytometry of THP-1 monocytes — unstained and in the presence of isotype control, anti-FcγRI/CD64, anti-FcγRII/CD32 (blue), and anti-FcγRIII/CD16 PE-conjugated antibodies. (**B** and **C**) ADCP facilitated by Cytogam under no-blocking and FcγR-blocking conditions. (**D**) ADCP across unblocked and FcγR-blocking conditions in all serum samples (*n* = 162). (**E**) ADCP across unblocked and FcγR-blocking conditions in maternal and cord blood sera from HCMV transmitting (red circles, *n* = 41) and non-transmitting (blue diamonds, *n* = 40) mother-infant dyads. (**F**) Ratio of FcγR-blocked/unblocked ADCP responses, calculated as (%AF647^+^ with FcR block)/(%AF647^+^ with no FcR block) in each serum sample (*n* = 162). (**G**) Ratio of FcγR-blocked/unblocked ADCP responses in transmitting versus non-transmitting dyads. Horizontal black bars denote median. *P* values for Mann-Whitney *U* test (**E** and **G**). *P < 0.05, **P < 0.01, ***P < 0.001.

To test our hypothesis that FcγRI and FcγRII engagement mediates enhanced ADCP in non-transmitting dyads, we measured ADCP in the presence and absence of FcγR-blocking antibodies. We found that the human monocyte cell line we used to measure ADCP, THP-1, had high expression of FcγRI and FcγRIIA but not FcγRIIB (Figure 7A and Supplemental Figure 11A). Blocking FcγRII with a pan anti-FcγRII and FcγRIIA-specific but not FcγRIIB-specific antibody inhibited ADCP (Figure 7, B and C, and Supplemental Figure 11B). Notably, blocking FcγRII at high Cytogam concentrations led to a much larger decrease in ADCP than at intermediate or low Cytogam levels (Figure 7, B and C, and Supplemental Figure 11B). These data indicate that FcγRI mediates ADCP most effectively at intermediate antibody levels and less efficiently at elevated antibody levels. Blocking FcγRI inhibited ADCP to a similar degree across Cytogam concentrations, suggesting that FcγRII-mediated ADCP was directly correlated with HCMV-specific antibody levels (Figure 7C). In maternal serum samples, blocking FcγRI only (median decrease, 40%), FcγRII only (median decrease, 70%), and FcγRI plus FcγRII (median decrease, 90%) inhibited ADCP to varying degrees (Figure 7, D–G). Non-transmitting dyads had significantly higher ADCP responses compared with transmitting dyads across all FcγR-blocking conditions (Figure 7E and Supplemental Figure 12), and FcγRI-mediated ADCP was particularly enhanced in non-transmitters (Figure 7G). Together, these findings suggest that ADCP mediated by FcγRI and to a lesser extent FcγRIIA is associated with protection against cCMV transmission.

## Discussion

Despite decades of research, protective immune responses against cCMV transmission have remained elusive, and there are no licensed vaccines or therapeutics to prevent cCMV (12). Using a case-control cohort of cord blood donor HCMV transmitting (*n* = 41) and non-transmitting (*n* = 40) mother-infant dyads, we identified Fc-mediated immunity and ADCP as potential correlates of protection against cCMV infection. These findings could guide future prospective studies on immune correlates against cCMV transmission and inform HCMV vaccine and immunotherapeutic development.

In our recent complementary study, we discovered that placental IgG transfer was modestly decreased in cCMV infection (43), prompting us to investigate HCMV-specific IgG transfer in HCMV transmitting pregnancies. Our finding that cCMV-infected infants had high levels of neutralizing and non-neutralizing HCMV-specific IgG suggests that reduced IgG transfer into the fetal circulation is not a risk factor for congenital infection. Low-avidity HCMV-specific IgG was enriched in the cord blood of infected infants, which has been proposed as a mechanism of antibody-dependent enhancement (52, 53). However, we speculate that this phenomenon is likely an indication of recent maternal primary infection or reinfection leading to the presence of an abundance of low-avidity antibodies early in pregnancy that were then transferred across the placenta. Together, our results further demonstrated that high-avidity HCMV-specific IgG in the maternal and fetal circulation is associated with protection against cCMV infection (20, 23, 43, 54).

HCMV antigen-specific IgG levels and neutralizing antibody titers have been correlated with protection against cCMV transmission in some studies (21, 24), yet these antibody responses were not associated with decreased transmission risk in our cohort. Our findings are consistent with a recent study in which Dorfman et al. also observed that higher maternal gB- and pentamer-specific IgG levels at delivery were associated with cCMV infection (25). Though higher HCMV-specific IgG levels were associated with increased transmission, antibody-dependent enhancement of infection is unlikely, given compelling epidemiological and experimental evidence that maternal antibodies help protect against congenital transmission (2, 55–57). Since both studies measured maternal HCMV-specific antibody responses at the delivery time point, we infer instead that elevated IgG levels and neutralizing antibody titers in transmitters are likely due to “boosting” from active HCMV infection or reactivation during pregnancy (25). This interpretation is bolstered by existing literature on cCMV immune correlates describing measurement of antibody responses at different time points in pregnancy. In maternal primary infection, Boppana et al. also identified higher gB-specific IgG titers in transmitting versus non-transmitting women at delivery (20). In contrast, Vanarsdall et al. found that seropositive transmitting and non-transmitting women had similar gB-, pentamer-, and gH/gL/gO-specific IgG levels and neutralization titers when measured in the first trimester (26). Moreover, Huang et al. recently demonstrated that higher pp150-specific IgG levels were associated with reduced cCMV transmission risk in HCMV-seropositive women when measured early in gestation (58). Together, these data suggest that elevated HCMV-specific IgG levels and neutralization titers at delivery are not causally associated with cCMV infection but correlated with transmission risk, because transmitting women are more likely to experience sustained viral replication during pregnancy.

Our study is the first to our knowledge to examine Fc-mediated antibody responses in cCMV transmission, and the finding that ADCP was associated with reduced cCMV infection could represent a step forward for the field. ADCP can defend against both cell-associated and cell-free virus through phagocytosis of virally infected cells or virus:IgG immune complexes (59, 60). Since HCMV infection primarily spreads cell-to-cell in vivo, it is logical that ADCP would be associated with protection against placental transmission (61). FcγRI and FcγRIIA, which we demonstrate mediate this ADCP, are highly expressed on maternal- and fetal-derived monocytes and macrophages at the maternal-fetal interface (62–64), so it is intriguing to consider whether these innate immune cells employ Fc-mediated functions against HCMV (39). Future studies should investigate whether ADCP or other Fc-mediated antibody functions protect against cCMV transmission systemically and/or at the maternal-fetal interface. Notably, different biophysical and biochemical properties of IgG including subclass and glycosylation also modify FcγR-binding affinity and downstream effector functions, so whether these Fc characteristics modulate cCMV transmission risk should also be explored (65–67). Together, our data suggest that enhanced phagocytosis, antiviral signaling, and/or antigen presentation following IgG binding to FcγRI and FcγRIIA may help protect against cCMV infection; however, the role of the inhibitory FcγRIIB remains unclear, as this receptor was not expressed on the monocytes we used to measure ADCP (47, 59, 68).

These findings can inform the development of vaccines and immunotherapeutics to prevent cCMV infection. Maternal treatment with HCMV hyperimmune globulin during primary infection to prevent congenital transmission has not been efficacious in two randomized clinical trials despite showing some efficacy in smaller observational studies (28–33). In our study, we observed that FcγRI-mediated ADCP was reduced at elevated Cytogam levels. Whether poor engagement of Fc-mediated immunity partially explains the limited efficacy of HCMV hyperimmune globulin is unknown but targeting certain Fc effector functions may improve polyclonal or monoclonal antibodies to prevent cCMV transmission.

Our results could have important implications for HCMV vaccine development beyond the context of congenital infection. The most successful HCMV vaccine to date was a gB subunit vaccine with an MF59 adjuvant that achieved approximately 50% efficacy, yet the antibody responses mediating this protection have remained elusive (35, 37, 38, 69). Neutralizing antibodies were poorly elicited by the gB/MF59 vaccine, leading researchers to hypothesize that non-neutralizing antibody responses mediated vaccine efficacy (34, 36, 38). Though ADCC responses were poorly stimulated in gB/MF59 vaccinees, ADCP responses were robustly induced (34, 36, 38). Moreover, there was a trend (β = –0.420, *P* = 0.120) toward ADCP being associated with protection against virus acquisition in our recent study of adolescent and postpartum gB/MF59 vaccinees (36). Notably, IgG binding to cell-associated gB, which we identified as an immune correlate of protection for these gB/MF59 vaccinees, was correlated with ADCP and FcγRI/FcγRIIA activation in our mother-infant cohort study (36). Taken together, these data suggest that Fc-mediated IgG responses against gB expressed on the surface of a virion or cell may protect against HCMV infection. Non-neutralizing antibody responses against other targets should also be explored, as non-structural HCMV antigens were recently identified as potent targets of ADCC (70). Cumulatively, these findings indicate that Fc-mediated and polyfunctional antibody responses against diverse HCMV antigens should be investigated as potential correlates of protection in HCMV infection and vaccination (51, 65, 66, 71).

Our study has several limitations. Due to the cross-sectional and retrospective nature of this cord blood bank donor cohort, we could not definitively identify maternal primary versus nonprimary HCMV infections or the timing of maternal infection. Because of this, caution is warranted in interpreting our results, since differences between transmitting and non-transmitting groups may be biased by a higher rate of primary infection and/or reinfection in transmitting cases. This limitation highlights the need for future prospective studies with longitudinal sampling throughout pregnancy to define protective antibody responses against cCMV infection in both maternal primary and nonprimary infection. We also did not have information on the HCMV strains infecting transmitting versus non-transmitting women and so could not assess whether maternal exposure to different HCMV strains was contributing to differences in antibody responses between groups. Placental biospecimens and maternal PBMCs were not collected, so we could not investigate placental infection or maternal cellular immune correlates of protection, even though CMV-specific T cell responses have been associated with reduced cCMV transmission (23, 27, 72). Since long-term clinical outcomes were unavailable, we could not assess whether antibody responses correlated with protection against long-term disease sequelae, though all infants were born without signs of cCMV infection. Statistical power was limited by small sample size (*n* = 41 HCMV transmitting dyads), yet this represents one of the largest US-based cohorts for assessing cCMV immune correlates to-date and one of the few studies without the confounder of maternal HIV coinfection (21, 23–26, 73). Our study focused on ADCP and did not measure other Fc effector functions — such as ADCC, antibody-dependent neutrophil phagocytosis (ADNP), or antibody-dependent complement deposition (ADCD) — due to limited sera sample volumes, but these Fc functions should be explored in future studies.

Development of an efficacious HCMV vaccine has been considered a “top-tier priority” by the US National Academy of Medicine for over 20 years, but identifying immune correlates of protection to inform vaccine development has proved challenging (74, 75). Our study suggests that eliciting HCMV-specific IgG that engages FcγRI/FcγRIIA and mediates non-neutralizing Fc effector functions such as ADCP may be an important immune response in the prevention of cCMV transmission and a promising new approach for HCMV vaccinology. It is hoped that these findings will guide future studies on correlates of protection against HCMV and inform the development of novel vaccines and therapeutics to prevent cCMV infection.

## Methods

### Study population.

We analyzed maternal and cord blood sera from 81 mother-infant dyads recruited from 2008 to 2017 as donors to the CCBB. The CCBB collects maternal and cord blood biospecimens at delivery, and mother-infant dyads were identified from over 29,000 CCBB donor records (Supplemental Figure 1). Maternal donors underwentinfectious diseases screening for HCMV, hepatitis B virus, syphilis, hepatitis C virus, HIV-1/2, HTLV I and II, Chagas disease, and West Nile virus. All mothers in our study were HCMV IgG seropositive and were negative for other infectious diseases. Cord blood plasma was screened by the CCBB for HCMV infection with a real-time PCR cobas AmpliPrep/TaqMan nucleic acid test (WHO HCMV reference standard, limit of quantification 147 IU/mL; ref. 76), and all cord blood units positive for HCMV underwent a second confirmatory PCR test.

Cases of cCMV infection were defined as mother-infant dyads with cord blood that screened positive for HCMV viremia at birth per PCR testing. “HCMV transmitting” cases with cCMV infection (*n* = 41) were matched to a target of 1 “HCMV non-transmitting” seropositive mother (*n* = 40) with no HCMV viremia detected in the cord blood (Supplemental Figure 1). Maternal HCMV IgG seropositivity and avidity were confirmed by a whole-virion HCMV ELISA, and HCMV IgM seropositivity was determined using a clinical diagnostic ELISA (Bio-Rad). Maternal serum was screened for HCMV DNAemia via quantitative PCR (qPCR). For qPCR, 150–200 μL maternal serum was ultracentrifuged; then DNA was extracted using the DNA QIAamp Kit (QIAGEN) and tested in duplicate using SybrSelect (Thermo Fisher Scientific) and 300 nM of forward and reverse primers to amplify the HCMV immediate early-1 (IE1) gene (24). HCMV viral loads were interpolated from an IE1 plasmid standard curve. Maternal serum was also screened for hypergammaglobulinemia (total IgG >15,000 mg/dL) via ELISA as previously described (43).

### Cell culture and HCMV virus growth.

Human retinal pigment epithelial cells (ARPE), human foreskin fibroblasts (HFFs), human embryonic kidney–293T (HEK293T) cells, and human monocytes (THP-1) were acquired from ATCC and cultured according to ATCC protocols. For differentiation, THP-1 cells were cultured in R10 medium with 100 nM PMA and incubated for 48 hours. HCMV strains TB40/E, AD169r, and Toledo were propagated as previously described (36, 77). HFFs (Toledo) or ARPEs (TB40E and AD169r) were plated in T175 flasks, rested overnight, and infected (MOI of 0.01) the next day once cells reached approximately 80% confluency. Infected HFFs or ARPEs were incubated for approximately 14 days until 90%–95% of cells showed a cytopathic effect. Harvested cells were either sonicated or subjected to multiple freeze-thaws and then centrifuged to pellet cells before the cell-associated “supernatant” was combined with collected cell-free supernatant. This combined cell-associated and cell-free virus stock was filtered (0.45 μm), then concentrated on a 20% sucrose cushion at 20,000 rpm (ca. 53,000*g*) in a Beckman SW28 rotor for 1.5 hours. The concentrated virus pellet was resuspended in HFF or ARPE growth medium (0.2 M sucrose), and the titer of concentrated virus stocks was determined with HFFs (Toledo) or ARPEs (TB40E and AD169r) by the limiting dilution technique in 96-well plates.

### Whole-virion HCMV IgG binding and avidity.

384-well ELISA plates were coated with 33 PFU/well TB40/E, 2700 PFU/well AD169r, or 1000 PFU/well Toledo virus (optimized PFU based on Cytogam binding; see Supplemental Figure 2); diluted in 0.1 M sodium bicarbonate buffer; then incubated overnight before blocking. Maternal and cord blood serum serial dilutions were tested starting at 1:30, plated in duplicate, then incubated before addition of HRP-conjugated goat anti–human IgG Fcγ (Jackson ImmunoResearch Laboratories Inc., 109-035-008). Plates were developed with tetramethylbenzidine (TMB) and peroxidase substrate (KPL), and OD at 450 nm was measured via SpectroMax (SPECTRO Analytical Instruments). HCMV-specific IgG concentrations were interpolated from the linear range of a 5-parameter HCMV-hyperimmune globulin (Cytogam) standard curve. HCMV binding to monoclonal IgG antibodies against gB (TRL-345, in-house), pentamer (TRL-310, in-house), pp65 (1-L-11, Thermo Fisher Scientific), and UL44 (M612460, Genway Bio) were also included for comparisons across strains. For determination of IgG avidity, duplicate wells were treated with 7 M urea or 1× PBS between the primary and secondary incubation steps, and relative avidity index (RAI) was calculated as (OD with urea)/(OD with PBS) × 100%. Duplicates with coefficients of variance (CVs) greater than 20% were repeated.

### HCMV glycoprotein–specific IgG binding and avidity.

A binding antibody multiplex assay (BAMA) was used to quantify HCMV glycoprotein–specific IgG binding and avidity (77, 78). HCMV gB ectodomain, pentamer complex, gH/gL/gO, gH/gL, pp28, pp150, and UL44 antigens were covalently coupled to intrinsically fluorescent beads (Bio-Rad). Maternal and cord blood sera were diluted at 1:500 (for gB ectodomain, pentamer complex, gH/gL/gO, and gH/gL) and at 1:25 (for pp28, pp150, and UL44) in assay diluent, plated in duplicate, then coincubated with antigen-coupled beads. Antigen-specific IgG binding was detected with mouse anti-human IgG–PE (SouthernBiotech), and MFI was acquired on a Bio-Plex 200 (Luminex). For determination of avidity, duplicate wells were incubated with sodium citric acid (pH 4.0) or 1× PBS (pH 7.4) between the primary and secondary incubations, and RAI was calculated as (MFI with sodium citric acid)/(MFI with PBS) × 100%. A serial dilution of HCMV-hyperimmuneglobulin (Cytogam) was included as a positive control, and the cutoff for positivity was determined by calculating the mean MFI of seronegative serum binding plus 3 SDs. Blank beads and wells were included to account for background signal. Duplicates with CVs greater than 25% were repeated.

### FcR binding by HCMV glycoprotein–specific IgG.

FcR binding by HCMV glycoprotein–specific IgG was measured using a modified BAMA (36, 79). Purified human FcR1a, FcR2a (clone H131), and FcR2b were produced by the Duke Human Vaccine Institute (DHVI) Protein Production Facility and biotinylated in-house. Maternal and cord blood sera were diluted at 1:500 (for gB ectodomain, pentamer complex, gH/gL/gO, and gH/gL) and at 1:25 (for pp28, pp150, and UL44) in assay diluent before incubation with HCMV antigen–coated beads, as above. Next, biotinylated human FcRs were complexed with streptavidin-PE (BD Biosciences), then coincubated with antibody-bound beads after washing. MFI was acquired on a Bio-Plex 200, and duplicates with CVs greater than 25% were repeated.

### Cell-associated HCMV gB IgG binding.

A gB-transfected cell-binding assay was used to measure IgG binding to cell-associated gB (36). HEK293T cells were cotransfected with DNA plasmids expressing GFP and full-length gB (Sino Biological) using the Effectene Transfection Kit (QIAGEN). After incubation at 37ºC for 48 hours, 200,000 live cells/well were plated into 96-well U-bottom plates, then centrifuged and washed before a 5-minute incubation in Human TruStain Fc Block (1:1000 dilution; BioLegend). In duplicate, cells were then coincubated with maternal and cord blood sera diluted 1:500 or with controls for 2 hours at 37°C. A serial dilution of HCMV-hyperimmuneglobulin (Cytogam) and a gB-specific monoclonal antibody (TRL-345, in-house) were included as positive controls, and seronegative serum samples were included as negative controls. Following incubation, cells were stained with Live/Dead Near-IR (1:1000; Invitrogen), then washed and stained with PE-conjugated goat anti-human IgG–Fc (1:200; SouthernBiotech). Last, cells were washed and fixed with 10% formalin. Events were acquired on an LSR II flow cytometer (BD Biosciences), and the percentage of PE-positive cells was calculated from the live, GFP-positive cell parent population using FlowJo (36). Unstained cells and single color–stained cells were included for setting gates and compensation. The cutoff for positivity was the mean signal of HCMV-seronegative samples plus 3 SDs. Duplicates with CVs greater than 50% were repeated.

### Neutralization.

HCMV neutralization was measured by high-throughput fluorescence bioimaging (36). Epithelial cells (ARPEs), fibroblasts (HFFs), or differentiated monocytes/macrophages (THP-1 cells) were plated in 384-well clear, flat-bottom plates and then incubated at 37°C overnight. To quantify neutralization, maternal and cord blood serum samples were diluted 1:10, followed by an 8-point serial dilution, and then coincubated with HCMV strain AD169r (MOI 2) or Toledo (MOI 1) for 2 hours at 37°C to allow immune complex formation. Virus-only wells and an 8-point HCMV-hyperimmuneglobulin (Cytogam) serial dilution were included as positive controls, while seronegative samples and no-virus wells were included as negative controls. After addition of the virus:serum mixture, cells were incubated at 37°C for 24–48 hours (depending on optimized conditions for each virus strain and cell type), then fixed in 10% formalin. To quantify HCMV infection, plates were stained with mouse anti–HCMV IE1 gene (1:500; Millipore, MAB810), followed by goat anti–mouse IgG–AF488 (1:500; Millipore), and cell nuclei were stained with DAPI (1:10,000; Thermo Fisher Scientific). After staining, plates were visualized with a Cellomics fluorescence plate reader (Supplemental Figure 5) to enumerate total cell count, infected cell count, and the percentage of infected cells in each well. Following image acquisition, neutralization titers corresponding to the dilution that resulting in a 50% reduction in percent infected cells (ID_50_) were calculated using interpolation in GraphPad Prism (Supplemental Figure 5). Samples containing wells with low cell counts and with duplicates with CVs greater than 50% were repeated.

### Whole-virion HCMV ADCP.

HCMV strains TB40/E, AD169r, and Toledo were conjugated to fluorochrome AF647 (Invitrogen) to measure ADCP (36). In brief, 2.0 × 10^6^ PFU TB40/E, 1.0 × 10^7^ PFU AD169r, or 1.0 × 10^7^ PFU Toledo virions were buffer exchanged with 1× PBS using a 100,000 kDa Amicon filter (Millipore) and conjugated to 10 μg AF647 *N*-hydroxysuccinimide ester reconstituted in DMSO during a 1-hour incubation with constant agitation. The conjugation reaction was quenched with 80 μL 1 M Tris-HCl (pH 8.0), and fluorescently labeled virus was diluted 25× in wash buffer. A serial dilution of HCMV-hyperimmuneglobulin (Cytogam) was included as a positive control, while seronegative serum samples and an anti-RSV monoclonal antibody (Synagis) were included as negative controls. In a 96-well plate, fluorescently labeled virus was coincubated with maternal sera, cord blood sera (1:10), or controls at 37°C for 2 hours to allow immune complex formation, before addition of 50,000 THP-1 cells per well. Plates were then centrifuged (1200*g*) at 4°C for 1 hour in a spinoculation step before a 1-hour incubation at 37°C to allow for phagocytosis. Next, cells were transferred to a 96-well U-bottom plate, washed, and fixed with 1% formalin. Events were acquired on an LSR II flow cytometer, and the percentage of AF647-positive cells was calculated from the live THP-1 monocyte cell parent population using FlowJo (gating strategy in Supplemental Figure 5). Unstained cells and single color–stained cells were included as controls for setting gates and compensation. The cutoff for positivity was the mean signal of HCMV-seronegative samples plus 3 SDs, and duplicates with CVs greater than 50% were repeated.

To measure FcγR expression, THP-1 cells were stained with anti–human CD64–PE (clone 10.1, eBioscience), CD32-PE (clone 6C4, eBioscience), CD32A–FITC (clone IV.3, StemCell Technologies), CD32B–APC (clone S18005H, BioLegend), CD16–PE (clone CB16, eBioscience), and Ig-PE isotype control (clone P3.6.2.8.1, eBioscience). Cells were fixed with 2% paraformaldehyde, acquired on an LSR II flow cytometer, and analyzed using FlowJo v10.7.2. For FcγR blocking, THP-1 cells were coincubated with purified anti–human CD64 (clone 10.1, BioLegend), CD32 (clone AT10, Bio-Rad), CD32A (clone IV.3, StemCell Technologies), or CD32B (clone S18005H, BioLegend) for 1.5 hours at 37°C prior to coincubation with virus:serum immune complexes. The remainder of the ADCP assay was performed as described above. An ADCP score to account for nonspecific background ADCP signal was calculated as follows: (%AF647-positive cells × AF647 MFI in serum sample)/(%AF647-positive cells × AF647 MFI in PBS control wells) × 100%.

### FcγR signaling assay.

HCMV-specific IgG signaling through FcγRs was measured using a previously published approach (48). Briefly, we used mouse BW thymoma cells stably expressing chimeric FcR-CD3ζ — which contains an extracellular human FcR and intracellular CD3ζ signaling domain — to quantify antiviral IgG activation of host FcγRs. To confirm FcγR expression, 0.5 × 10^6^ BW cells were added to each well in a 96-well plate. The nontransfected parental BW cell line and BW cell lines expressing human CD64 (FcR1a), CD32a (FcR2a), or CD32b (FcR2b) were stained for surface expression of human FcγRs with 5 μL anti–human CD64–PE (clone 10.1, eBioscience), 5 μL anti–human CD32–PE (clone 6C4, eBioscience), 5 μL CD16-PE (clone CB16, eBioscience), and 5 μL anti–human Ig–PE isotype control (clone P3.6.2.8.1, eBioscience); then cells were fixed with 2% paraformaldehyde. Events were acquired on an LSR II flow cytometer and analyzed using FlowJo v10.7.2.

To quantify FcγR activation, 96-well plates were coated with 20,000 PFU/well HCMV strain AD169r and incubated at 4°C overnight. After coating, plates were washed with assay buffer (1× PBS + 1% FBS), then blocked with buffer at room temperature for 1 hour. After blocking, HCMV-coated plates were coincubated with maternal or cord blood serum diluted 1:10 in BW cell media at 37°C for 1 hour. HCMV-hyperimmuneglobulin (Cytogam), seronegative, and no-antibody conditions were included as controls. Following immune complex formation, plates were washed with BW media before addition of 100,000 FcR1a-, FcR2a-, or FcR2b-expressing BW cells per well. In separate wells, the parental (nontransfected) BW cells were added as a negative control. Cells were incubated at 37°C with immune complex–coated plates for 20 hours before being transferred to V-bottom plates and pelleted (1200 rpm [ca. 250*g*]). Cell supernatants were harvested, and mouse IL-2 (mIL-2) levels in culture supernatants were measured using ELISA. For the mIL-2 ELISA, 384-well plates were coated with 3 μg/mL purified rat anti–mouse IL-2 (BD Biosciences) and incubated at 4°C overnight before blocking. Purified mIL-2 and BW cell culture supernatants were added in duplicate and incubated for 1 hour at room temperature. After primary incubation, plates were incubated with rat anti–mIL-2 conjugated to biotin (1:2000; BD Biosciences) followed by streptavidin-HRP (1:8000) for 1 hour and 30 minutes, respectively. Plates were developed with TMB/KPL, then OD at 450 nm was measured via SpectroMax, and mIL-2 concentrations were interpolated from a 5-parameter mIL-2 standard curve using GraphPad Prism. Duplicates with CVs greater than 30% were repeated.

### Statistics.

All primary raw and analyzed data underwent independent data quality control by a second laboratory member prior to statistical analysis and inclusion in the study. For all analyses, interpolated antibody or cytokine concentrations, MFI values, and neutralization titers were log transformed to normalize the data distribution. Antibody responses below the limit of detection were set equal to the limit of detection for statistical analyses. To assess differences between HCMV transmitting and non-transmitting mother-infant dyads, immune variables were compared between groups using Mann-Whitney *U*/Wilcoxon’s rank-sum and within dyads using Wilcoxon’s signed rank tests. Statistical significance was defined a priori as *P* < 0.05 with a 2-tailed test and a Benjamini-Hochberg correction for multiple comparisons. For the primary immune correlate analysis, 13 predefined maternal humoral immune variables were included for univariate logistic regression analysis. Using the 13 predefined immune variables from our primary immune correlate analysis, LASSO regression analysis was performed using the caret R package, and 5-fold cross-validation with 5 repeats was performed. For the LASSO regression, the cohort was randomly split into 2 independent data sets, which were used for training of the LASSO model and testing the predictive performance of the model, respectively. To test the random prediction rate of the trained LASSO model, class lab permutation was performed on the data set, and model accuracy characteristics were assessed. All statistical analyses were completed in R v4.1.1 and GraphPad Prism v9.1. The PCA plots were rendered using ggplot2, and correlation matrices were plotted using the corrplot package in R. All other figures were generated using GraphPad Prism.

### Study approval.

Approval was obtained from Duke University’s Institutional Review Board (Pro00089256) to use deidentified clinical data and biospecimens provided by the CCBB. No patients were prospectively recruited for this study, and all samples were acquired retrospectively from the CCBB biorepository from donors who had previously provided written consent for banked biospecimens to be used for research.

## Author contributions

ECS, JAJ, JHH, KMW, and SRP designed the research study. ECS, IGM, CP, MJH, SJB, and HW conducted the experiments and acquired the data. ECS, IGM, and SJB completed the primary data analysis. CEW and ECS completed the statistical analyses with oversight from KMW. SRP and KMW acquired funding for the study. JK provided the biospecimens and clinical data for the study. ECS wrote the primary draft of the manuscript. ECS, IGM, CP, JAJ, CEW, SJB, MJH, HW, JHH, JK, GGF, KMW, and SRP contributed to writing and editing the manuscript.

## Supplementary Material

Supplemental data

## Figures and Tables

**Table 1:**
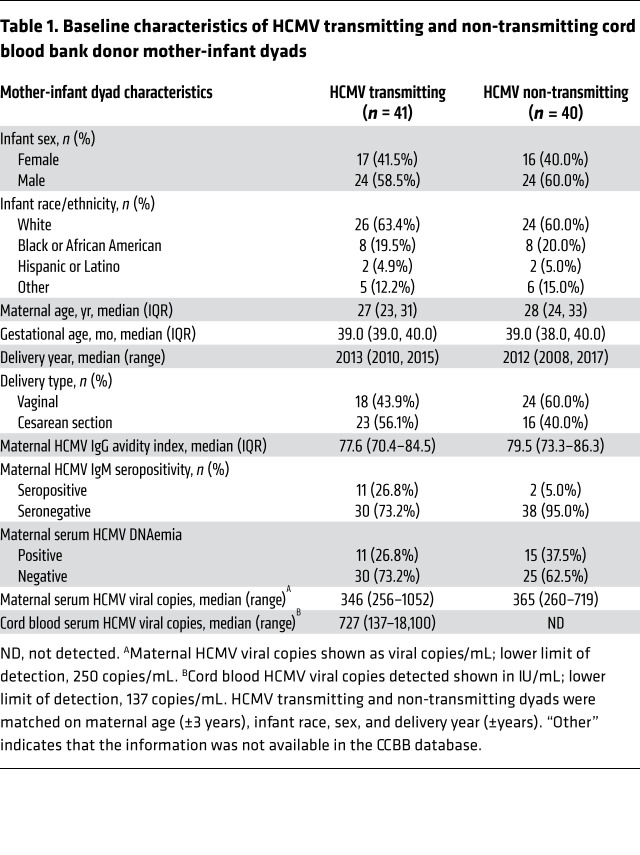
Baseline characteristics of HCMV transmitting and non-transmitting cord blood bank donor mother-infant dyads

**Table 2:**
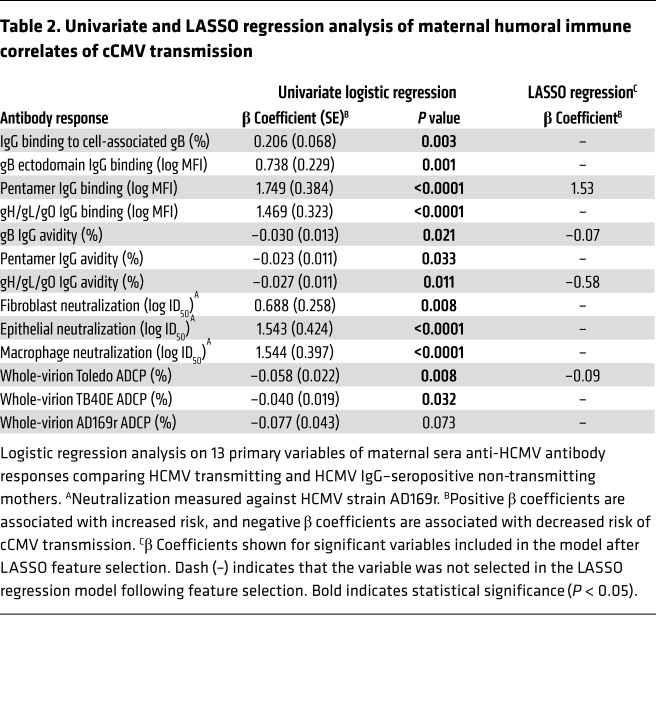
Univariate and LASSO regression analysis of maternal humoral immune correlates of cCMV transmission

## Version 1 06/28/2022

In-Press Preview

## Version 2 08/15/2022

Electronic publication
