# Supplementary material for: Maternal Fc-mediated non-neutralizing antibody responses correlate with protection against congenital human cytomegalovirus infection
Source: J Clin Invest. 2022 Aug 15;132(16):e156827. doi: 10.1172/JCI156827 (PMC9374380; doi:10.1172/JCI156827)
Supplement: Supplemental data [file jci-132-156827-s014.pdf]

Supplementary Figure 1. Identification of HCMV transmitting and non-transmitting mother-infant dyads from the Carolinas Cord Blood Bank (CCBB) biorepository.

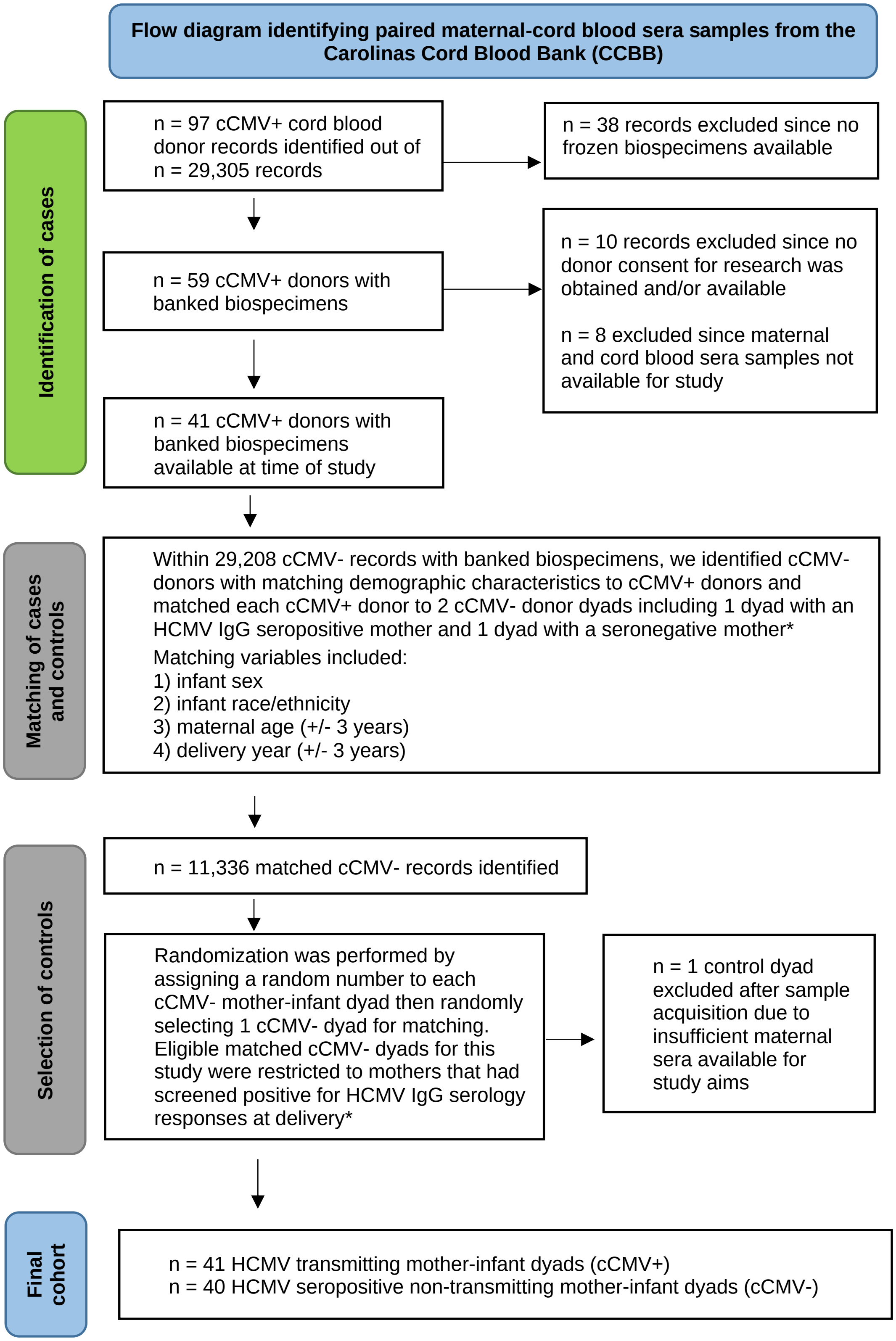

cCMV = congenital HCMV infection
cCMV+ = positive HCMV PCR cord blood screening at birth
cCMV- = negative HCMV PCR cord blood screening at birth
*Initial HCMV serology screening performed at time of donation by the American Red Cross in Charlotte, N.C.

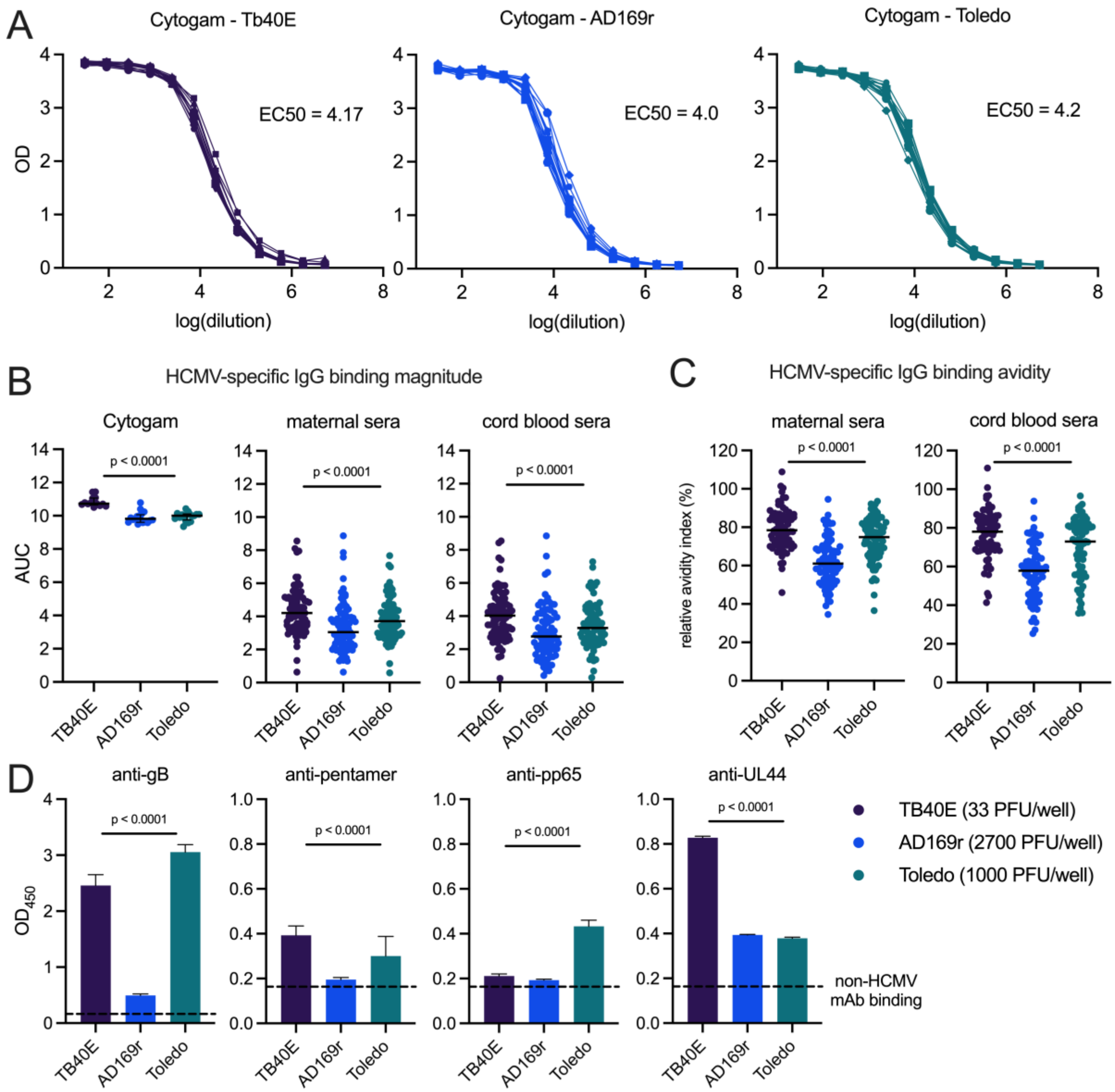

Supplementary Figure 2. Characterization of IgG binding to HCMV strains TB40E, AD169r and Toledo. HCMV-specific IgG levels against HCMV strains TB40E, AD169r, and Toledo were measured using enzyme-linked immunosorbent assay (ELISA). The number of plaque-forming units (PFUs) for each viral strain was optimized to achieve comparable binding to Cytogam. (A) Optical density (OD) values of the Cytogam standard curve binding to TB40E (33 PFU/well), AD169r (2700 PFU/well), and Toledo (1000 PFU/well). (B) Area under the curve (AUC) of Cytogam, maternal (n=81), and cord blood (n=81) sera sample IgG binding to TB40E, AD169r, and Toledo. (C) Avidity of maternal and cord blood sera sample IgG binding to TB40E, AD169r, and Toledo. (D) OD values of monoclonal antibody (mAb) IgG binding to TB40E, AD169r, and Toledo. Black bars denote median. EC50 values interpolated from 5-parameter standard curve. P values reported for one-way ANOVA test.

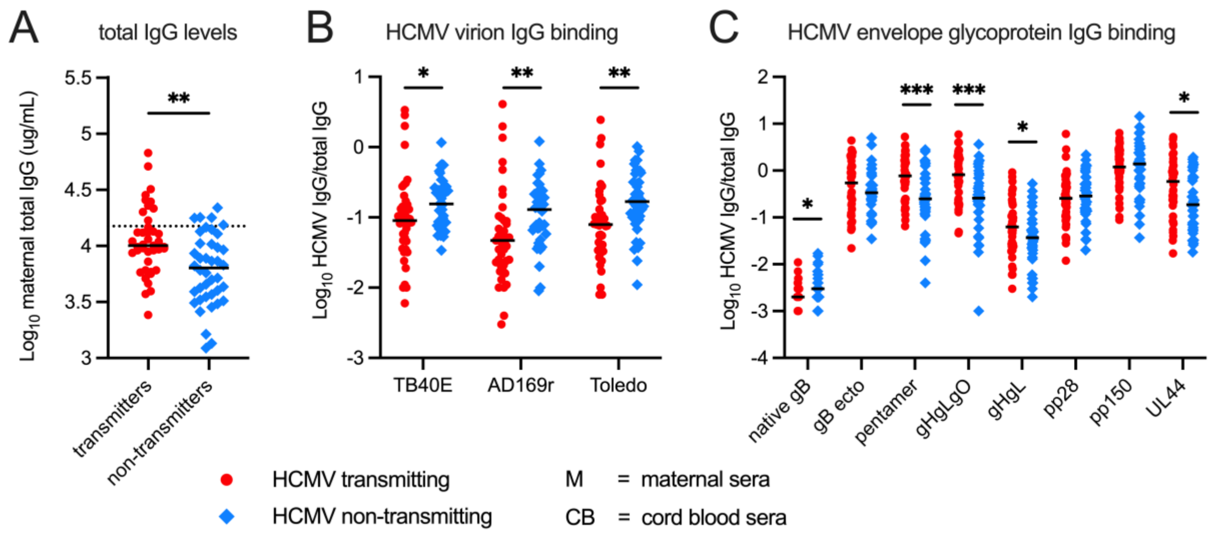

Supplementary Figure 3. Maternal HCMV-specific IgG binding corrected for total IgG levels. Total IgG levels and HCMV-specific IgG binding were quantified using ELISA. (A) Maternal sera total IgG levels in HCMV transmitting (red circles, n = 41) versus non-transmitting (blue squares, n = 40) mothers. Dotted line indicates cut-off for hypergammaglobulinemia (total IgG concentration >15,000 mg/dL). (B-C) HCMV-specific IgG binding was corrected for total IgG levels by taking a ratio of HCMV-specific IgG/total IgG binding and ratios were compared in HCMV transmitting versus non-transmitting mothers. Black bars denote median. P values reported for Mann-Whitney U test (panels A, B, and C). * P < 0.05, ** P < 0.01, *** P < 0.001.

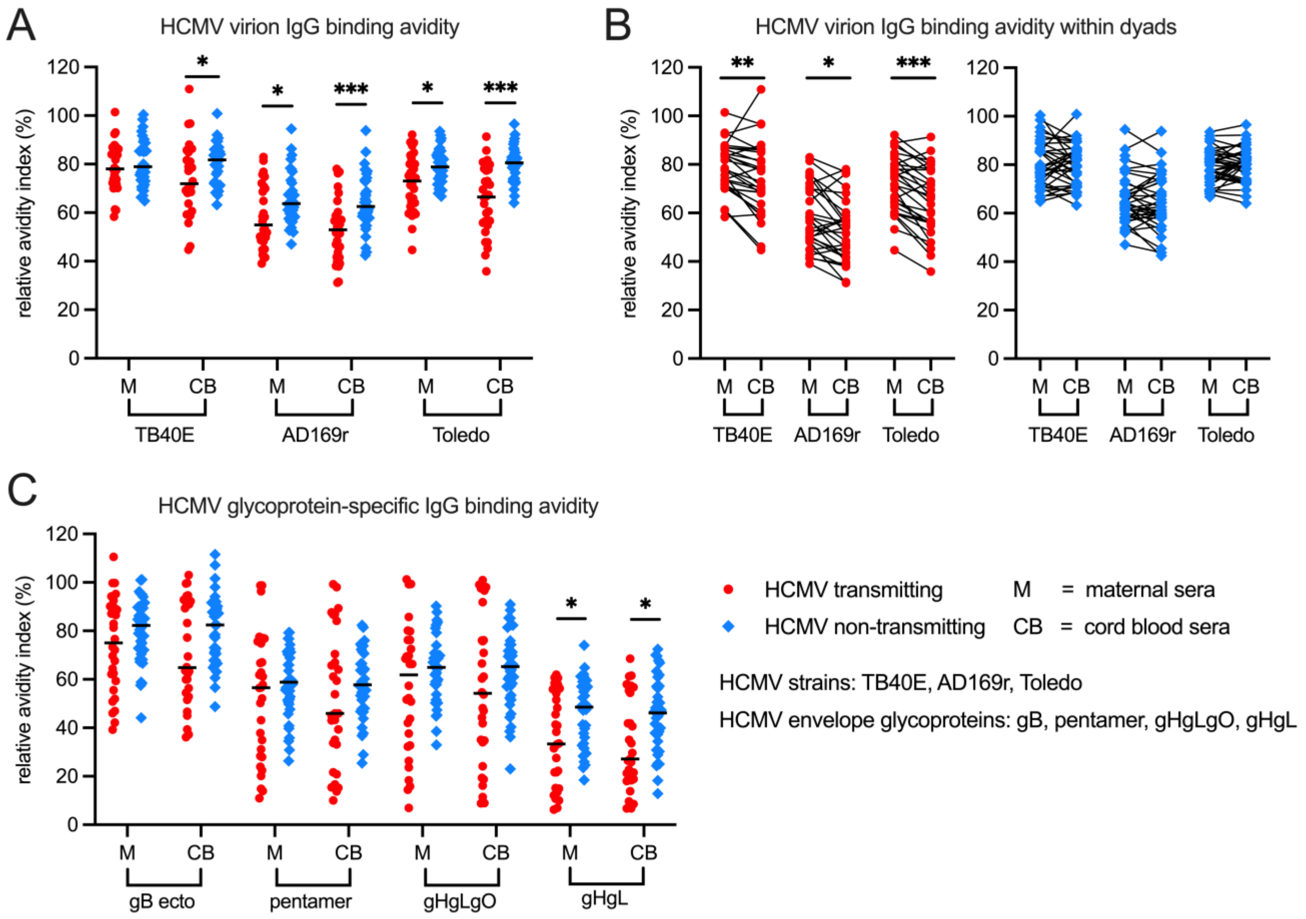

Supplementary Figure 4. HCMV-specific IgG binding avidity remains increased in non-transmitting versus transmitting mother-infant dyads when excluding mothers with detectable HCMV-specific IgM. HCMV-specific IgG binding avidity against HCMV strains TB40E, AD169r, and Toledo were measured using whole virion ELISA with an additional dissociation step using urea and relative avidity index (RAI) was calculated as (OD with urea/OD without urea)X100%. HCMV glycoprotein-specific IgG binding avidity was measured using a binding antibody multiplex assay with an additional dissociation step with sodium citrate buffer (pH = 4) and RAI was calculated as (MFI with sodium citrate/MFI with 1X PBS)X100%. In a sensitivity analysis excluding dyads where mothers screened positive for HCMV-specific IgM responses, IgG binding avidity in maternal (M) and cord blood (CB) sera was compared between and within a subset of transmitting (red circles, n = 30) and non-transmitting (blue squares, n = 38) mother-infant dyads. (A-B) Whole virus HCMV-specific IgG binding avidities (A) in transmitting versus non-transmitting dyads and (B) within paired maternal and cord blood sera. (C) HCMV glycoprotein-specific IgG binding avidities in transmitting versus non-transmitting dyads. gB ecto = gB ectodomain. Black bars denote median. FDR-corrected P values for Mann-Whitney U test (panels A and C) or Wilcoxon signed-rank test (panel B). * P < 0.05, ** P < 0.01, *** P < 0.001.

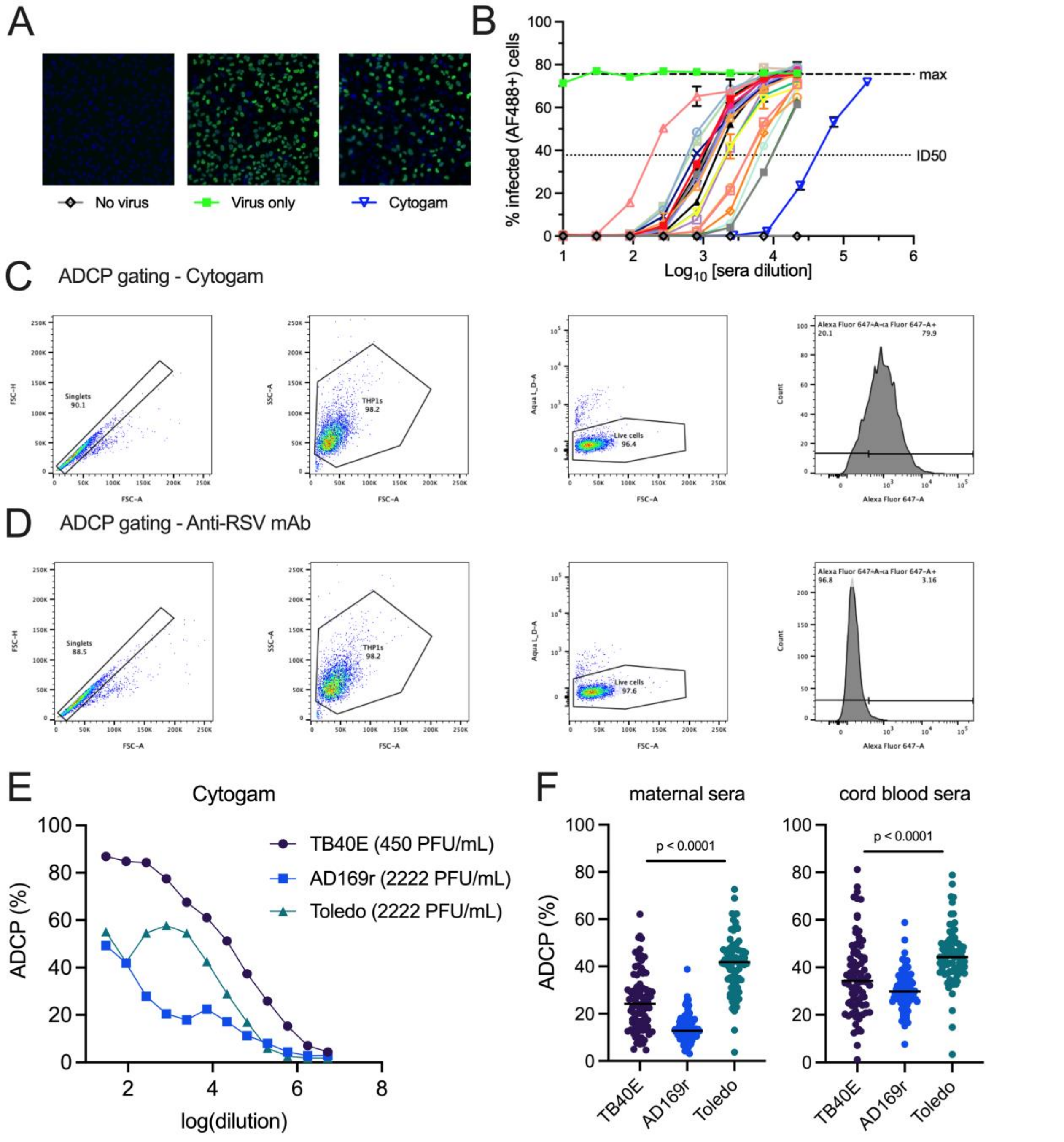

Supplementary Figure 5. HCMV neutralization and antibody-dependent cellular phagocytosis (ADCP) assay methods. Neutralization was measured by HCMV IE1 staining with AF488 and antibody titers were calculated as the inhibitory dilution 50 (ID50), equivalent to the sera dilution that inhibited 50% of the max infection in virus only wells. Infected cell percentage was calculated as a proportion of AF488 positive cells out of total DAPI+ cells per well. (A) IE1 staining (green) and DAPI staining (blue) of epithelial (ARPE) cells infected with HCMV AD169r strain showing representative no virus, virus only, and virus plus Cytogam treated wells. (B) Plot showing how ID50 values were interpolated from sera serial dilutions. (C-D) Flow cytometry gating strategy for ADCP assay showing gating for (C) Pavalizumab, a non-HCMV-specific monoclonal antibody (mAb) against respiratory syncytial virus (RSV) F protein, (negative control) and (D) Cytogam (positive control). (E) Cytogam ADCP responses across HCMV strains. (F) ADCP responses across HCMV strains in maternal (n=81) and cord blood (n=81) sera samples. Black bars denote median. ID50 values interpolated from 5-parameter standard curve. P values reported for one-way ANOVA test (panel F).

Supplementary Table 1. Univariate logistic regression analysis of maternal humoral immune correlates of congenital HCMV transmission adjusted for potential confounders.

	Univariate adjusted for maternal total IgG		Univariate corrected for maternal HCMV-specific IgM status	
**Antibody response**	**β [b]**	***P* value[c]**	**β [b]**	***P* value[c]**
**IgG binding to cell-associated gB (%)**	0.187	**0.007**	0.234	**0.003**
**gB ectodomain IgG binding (Log MFI)**	0.653	**0.006**	0.797	**0.001**
**pentamer IgG binding (Log MFI)**	1.712	**<0.0001**	1.863	**<0.0001**
**gHgLgO IgG binding (Log MFI)**	1.431	**<0.0001**	1.498	**<0.0001**
**gB IgG avidity (%)**	-0.027	**0.049**	-0.019	0.183
**pentamer IgG avidity (%)**	-0.018	0.106	-0.015	0.211
**gHgLgO IgG avidity (%)**	-0.022	0.050	-0.019	0.092
**Fibroblast neutralization (Log ID50)[a]**	0.536	**0.042**	0.829	**0.003**
**Epithelial neutralization (Log ID50)[a]**	1.328	**0.002**	1.490	**0.001**
**THP1 neutralization (Log ID50)[a]**	1.533	**<0.0001**	1.487	**<0.0001**
**Whole virion Toledo ADCP (%)**	-0.044	**0.046**	-0.048	**0.035**
**Whole virion TB40E ADCP (%)**	-0.023	0.254	-0.027	0.167
**Whole virion AD169r ADCP (%)**	-0.032	0.475	-0.059	0.182

[a] Neutralization measured against HCMV strain AD169r

[b] Positive beta coefficients are associated with increased risk and negative beta coefficients are associated with decreased risk of congenital HCMV transmission

[c] Bold indicates statistical significance (*P* value <0.05)

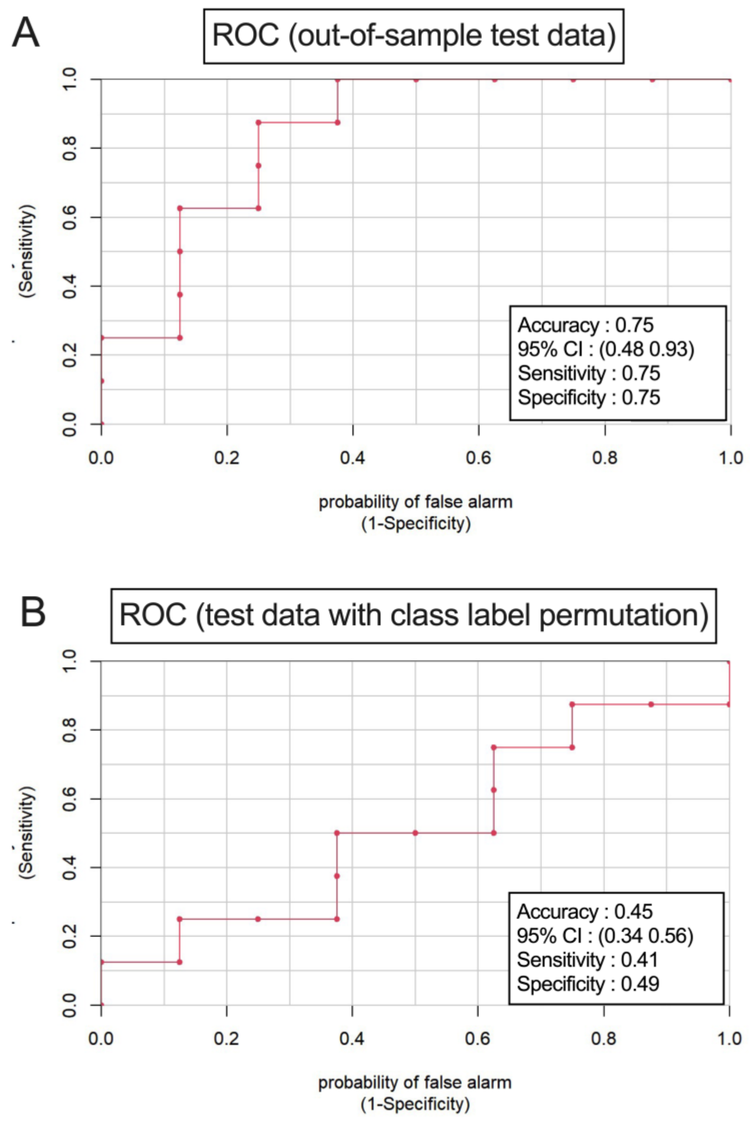

Supplementary Figure 6. ROC curves from LASSO model. Receiver operator characteristic (ROC) curves and model accuracy shown for trained LASSO model (5-fold cross validation with 5 repeats). (A) ROC curve and model accuracy for true out-of-sample test data and (B) ROC curve and model accuracy for data following class label permutation to simulate random prediction accuracy.

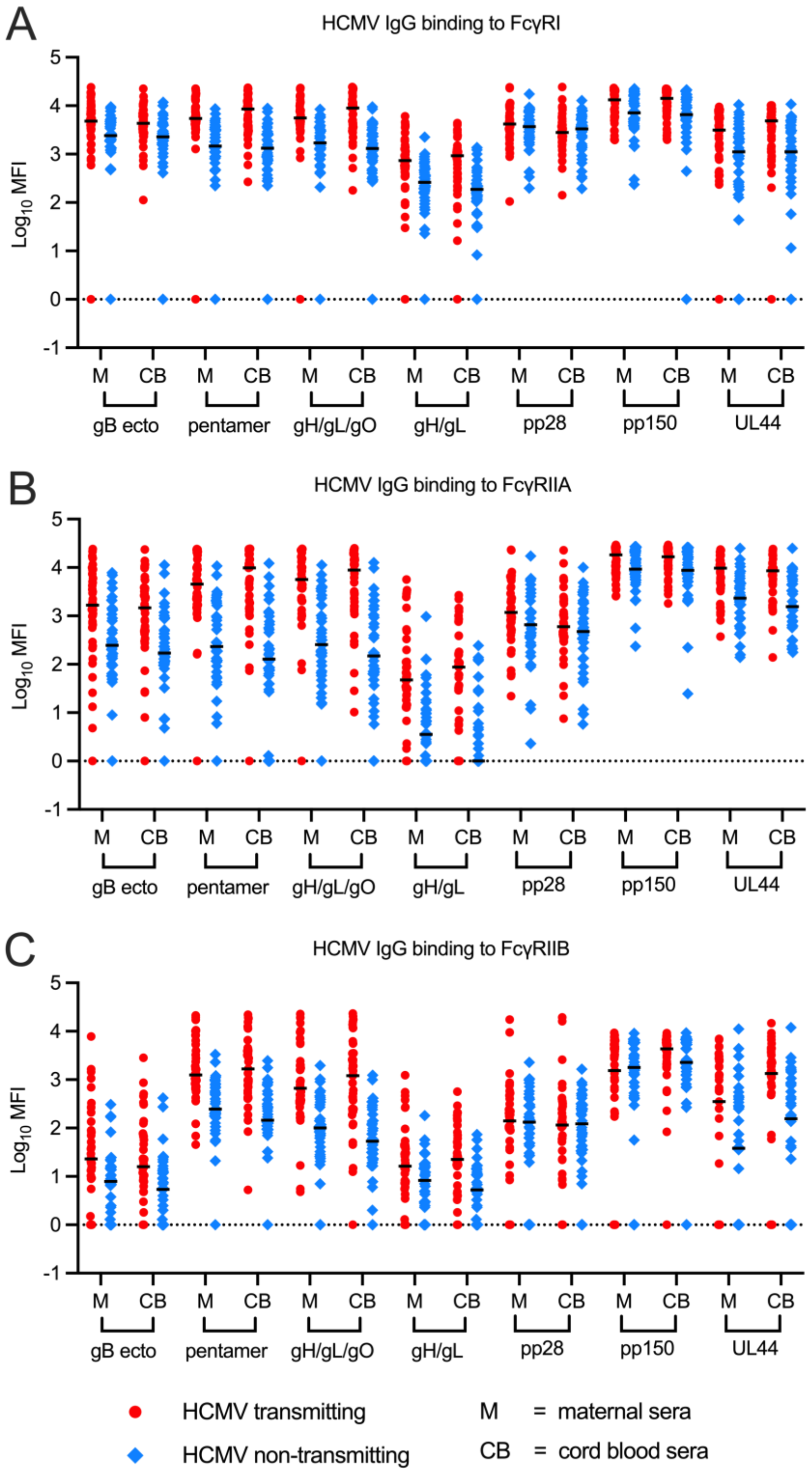

Supplementary Figure 7. Non-normalized HCMV antigen-specific IgG binding to Fc receptors in transmitting versus non-transmitting dyads. HCMV antigen-specific IgG binding to (A) FcγRI/CD64, (B) FcγRIIA/CD32A (high-affinity H131 variant) and (C) FcγRIIB/CD32B in transmitting (red circles, n = 41) and non-transmitting (blue squares, n = 40) mother-infant dyads. Black bars denote median. Dotted line indicates lower limit of detection. No statistical comparisons made.

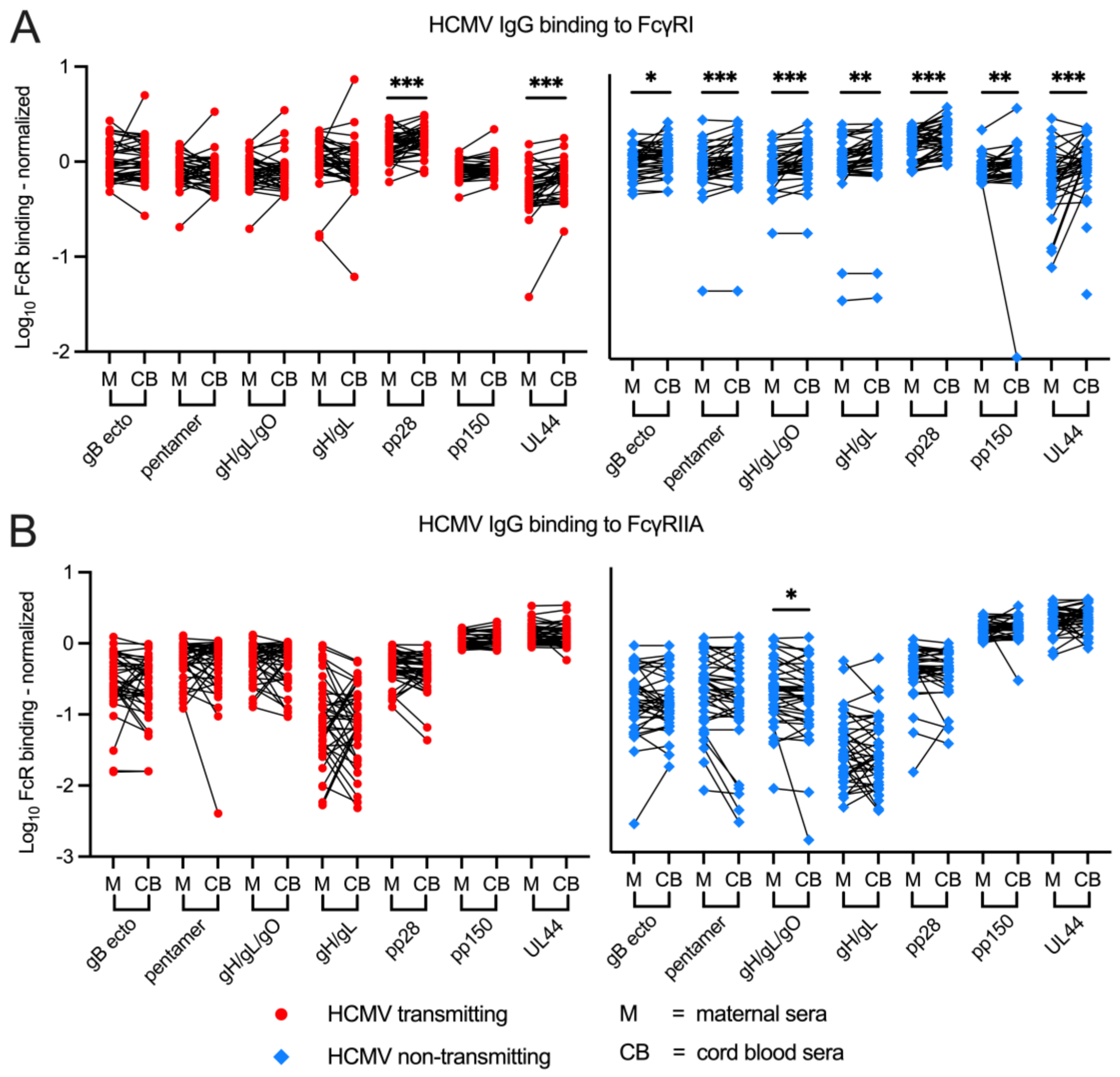

Supplementary Figure 8. Normalized HCMV antigen-specific IgG binding to Fc receptors within transmitting and non-transmitting mother-infant dyads. HCMV antigen-specific IgG binding to Fcγ receptors was measured using a Luminex-based binding antibody multiplex assay with a biotinylated FcγR and streptavidin-PE detection antibody. HCMV antigen-specific IgG binding to host FcγRs was normalized as a ratio of total antigen-specific IgG binding and was compared within transmitting (red circles, n = 41) and non-transmitting (blue squares, n = 40) mother-infant dyads. (A-B) HCMV-specific IgG binding to FcγRI and (C-D) FcγRIIA (high affinity H131 variant). gB ecto = gB ectodomain. Black bars denote median. FDR-corrected P values reported for Wilcoxon signed-rank test (panels A and B). * P < 0.05, ** P < 0.01, *** P < 0.001.

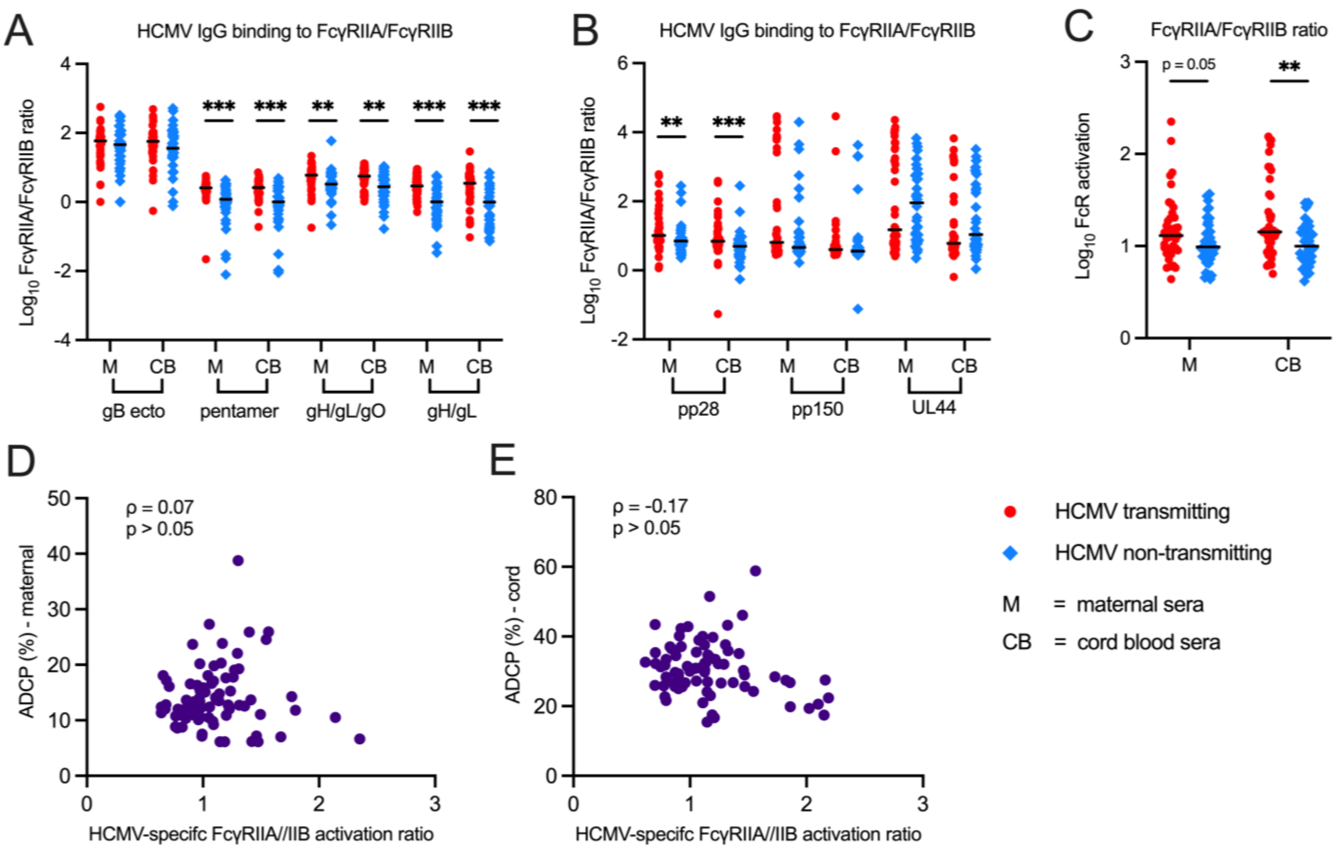

Supplementary Figure 9. Ratio of FcγRIIA to FcγRIIB engagement does not correlate with antibody-dependent cellular phagocytosis (ADCP) of HCMV. Ratios of activating FcγRIIA to inhibitory FcγRIIB engagement in maternal (M) and cord blood (CB) sera from HCMV transmitting (red circles, n = 41) and non-transmitting (blue squares, n = 40) mother-infant dyads. (A-B) Ratio of HCMV-specific IgG binding to FcγRIIA/FcγRIIB. (C) Ratio of HCMV-specific IgG activation of FcγRIIA/FcγRIIB as measured by BW reporter cell assay. (D-E) Spearman correlations between ADCP and ratio of FcγRIIA/FcγRIIB activation as measured by BW reporter cell assay. Black bars denote median. FDR-corrected P values for Mann-Whitney U test (panels A-C) or Spearman rank correlation (panels D and E). * $P < 0.05$, ** $P < 0.01$, *** $P < 0.001$.

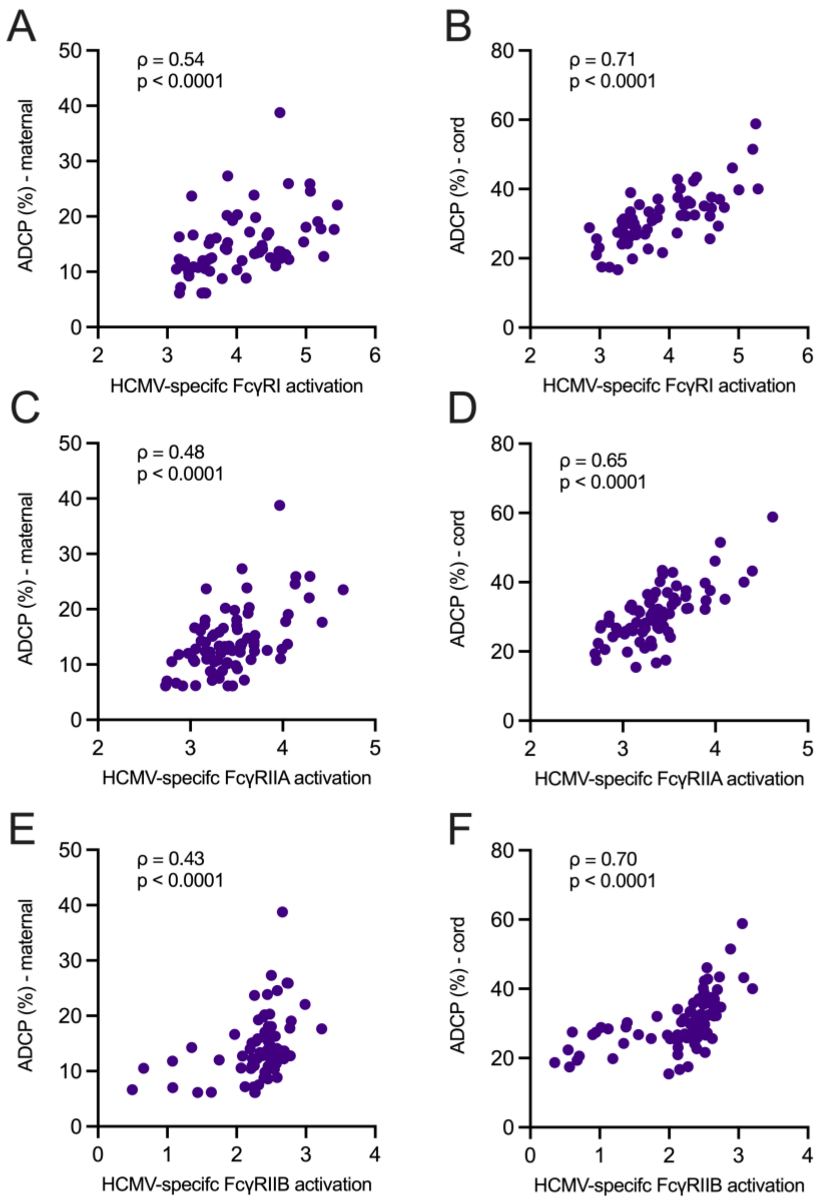

Supplementary Figure 10. Correlation between HCMV-specific IgG FcγR activation and ADCP responses. Spearman correlations between ADCP and HCMV-specific IgG activation of (A-B) FcγRI, (C-D) FcγRIIA, and (E-F) FcγRIIB as measured by BW cell reporter assay in maternal (n=81) and cord blood (n=81) sera.

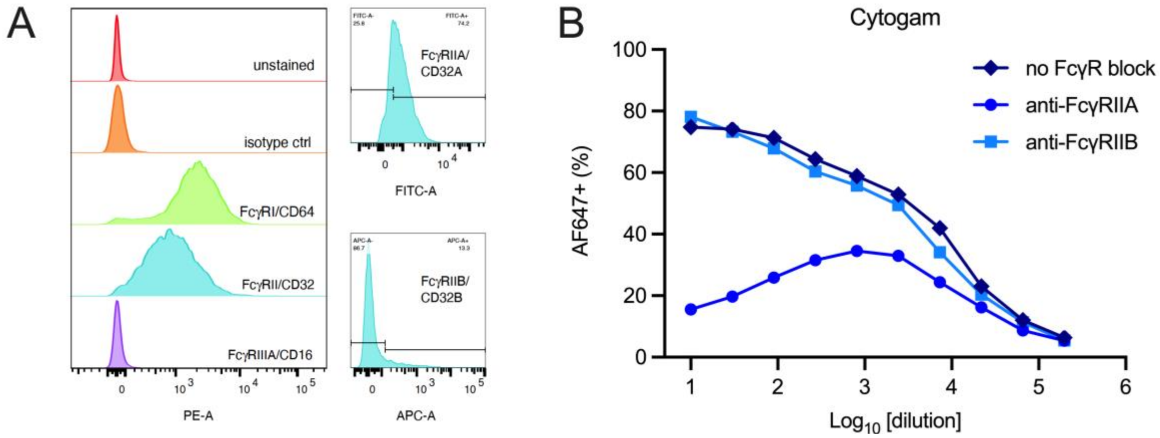

Supplementary Figure 11. Inhibitory receptor FcγRIIB does not impact antibody-dependent cellular phagocytosis (ADCP) of HCMV by human THP1 monocytes. ADCP of AF647 fluorophore-conjugated HCMV virions (Toledo strain) by THP1 monocytes was quantified using a flow-based assay and calculated as percentage AF647 positive cells. In blocking experiments, THP1 monocytes were pre-incubated with FcγR blocking antibodies for 90 minutes prior to co-incubating virus:sera immune complexes with THP1 monocytes. (A) Flow cytometry of THP1 monocytes including unstained THP1s (red), isotype control (orange), anti-FcγRI/CD64 (light green), anti-FcγRII/CD32 (blue), and anti-FcγRIII/CD16 (purple) PE-conjugated antibodies as well as anti-FcγRIIA (FITC-conjugated) and anti-FcγRIIIB (APC-conjugated) specific monoclonal antibodies (blue). (B) ADCP facilitated by Cytogam under no blocking, FcγRIIA blocking and FcγRIIB blocking conditions.

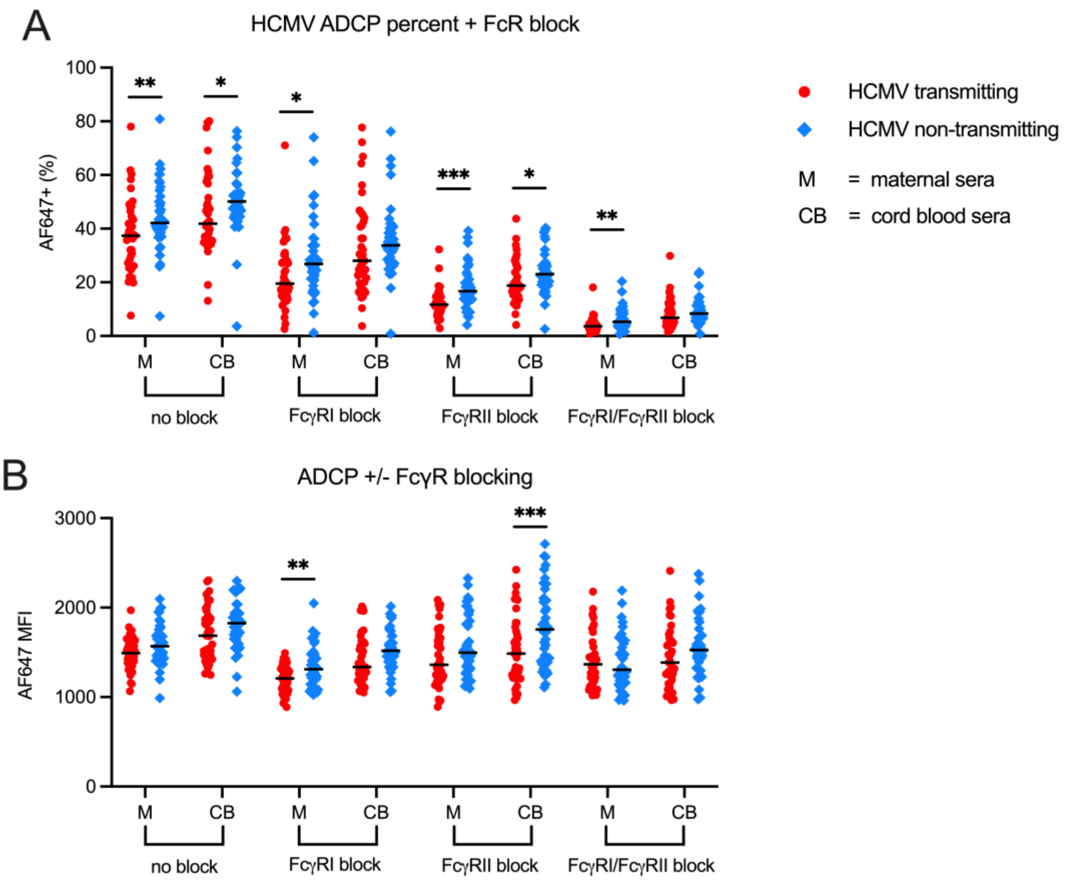

Supplementary Figure 12. Antibody dependent cellular phagocytosis (ADCP) of HCMV under unblocked and FcγR blocked conditions. ADCP of AF647 fluorophore-conjugated HCMV virions (Toledo strain) by THP1 monocytes was quantified using a flow-based assay and calculated as percentage AF647 positive cells. In blocking experiments, THP1 monocytes were pre-incubated with FcγR blocking antibodies for 90 minutes prior to co-incubating virus:sera immune complexes with THP1 monocytes. ADCP responses under unblocked and blocked conditions were compared in maternal (M) and cord blood (CB) sera from HCMV transmitting (red circles, n = 41) versus non-transmitting (blue squares, n = 40) mother-infant dyads. ADCP responses are expressed as (A) percentage AF647+ cells and (B) AF647 MFI. Black bars denote median. P values for Mann-Whitney U test (panels A-B). * P < 0.05, ** P < 0.01, *** P < 0.001.
